# A Review of Modifications of Quinoline Antimalarials: Mefloquine and (hydroxy)Chloroquine

**DOI:** 10.3390/molecules27031003

**Published:** 2022-02-02

**Authors:** Dawid J. Kucharski, Michalina K. Jaszczak, Przemysław J. Boratyński

**Affiliations:** Department of Organic and Medicinal Chemistry, Wrocław University of Technology, Wyspiańskiego 27, 50-370 Wrocław, Poland; dawid.kucharski@pwr.edu.pl (D.J.K.); 246137@student.pwr.edu.pl (M.K.J.)

**Keywords:** mefloquine, chloroquine, hydroxychloroquine, late-stage modification, semisynthesis, derivatization, quinoline

## Abstract

Late-stage modification of drug molecules is a fast method to introduce diversity into the already biologically active scaffold. A notable number of analogs of mefloquine, chloroquine, and hydroxychloroquine have been synthesized, starting from the readily available active pharmaceutical ingredient (API). In the current review, all the modifications sites and reactivity types are summarized and provide insight into the chemistry of these molecules. The approaches include the introduction of simple groups and functionalities. Coupling to other drugs, polymers, or carriers afforded hybrid compounds or conjugates with either easily hydrolyzable or more chemically inert bonds. The utility of some of the compounds was tested in antiprotozoal, antibacterial, and antiproliferative assays, as well as in enantiodifferentiation experiments.

## 1. Introduction

**Figure 1:**
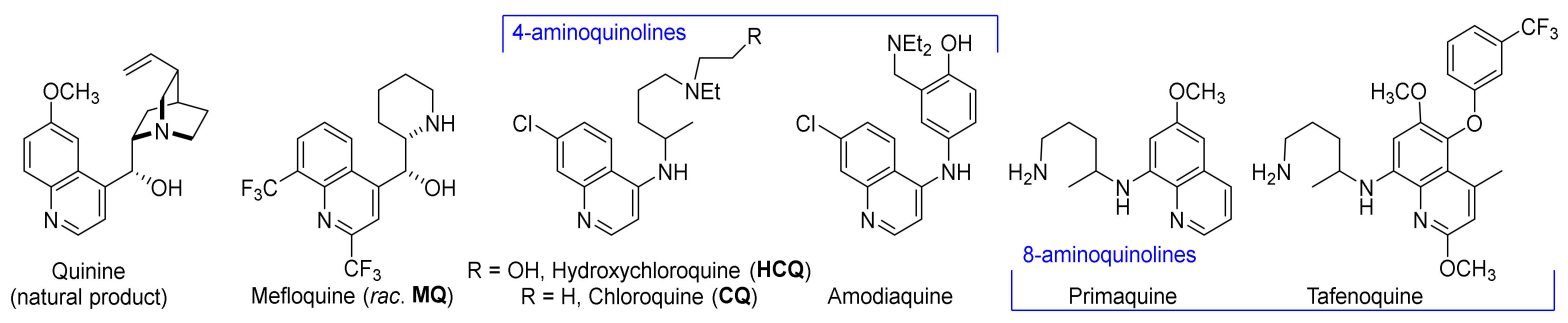
Quinoline antimalarials.

Malaria is an infectious disease, which is responsible for more than 600,000 deaths annually, mainly in tropical areas [1]. It is transmitted by certain *Anopheles* mosquitos and caused by *Plasmodium* parasites such as *P. knowlesi*, *P. malariae*, *P. ovale*, *P. vivax*, and *P. falciparum* [2]. The last species is the most life-threatening [1,3,4,5]. People suffering from malaria experience high fever, headaches, nausea, sweat, fatigue, diarrhea, and anemia [1]. The first effective drug against malaria was the *Cinchona* bark, which contains quinine. While the use of quinine in therapy is rare now, a number of quinoline drugs have been developed to fight malaria (Figure 1). Some of them are very close analogues of quinine, like mefloquine, others fall into 4-aminoquinoline (e.g., chloroquine) and 8-aminoquinoline families (e.g., primaquine). They, in combination with the non-quinoline drug artemisinin, are extensively used. Apart from antiparasitic effects, these medicines exhibit a plethora of biological activities including antibiotic, antitumor, and anti-inflammatory effects [1,2,3,4]. The most modern antimalarial medicine, tafenoquine, is an analog of much older primaquine. This exemplifies that the modification of one drug molecule can lead to a new effective analogous drug.

Two general approaches can be adopted. The universal one is to prepare each analog by a de novo synthesis (bottom-up approach). The other is to use an already available natural product or an industrially available active pharmaceutical ingredient (API) and perform further transformations. Such method referred to as semi-synthesis, late-stage modification, or top-down approach, has the advantage of usually being short. Sometimes it is a single-step process [6,7]. In the case of *Cinchona* alkaloids, this approach has been used to make analogs that are effective chiral catalysts or show promising antibiotic properties [8,9,10]. Unlike the natural products [8,9] or the de novo synthesized analogs [11,12,13,14], there is no comprehensive review on the late-stage modification of other quinoline drugs. Here we gathered transformations performed on three drugs resembling quinine: mefloquine (**MQ**), chloroquine (**CQ**), and hydroxychloroquine (**HCQ**). Whenever the term ‘mefloquine’ is used, it refers to the *erythro* enantiomer.

## 2. Mefloquine (MQ)

**Scheme 1:**
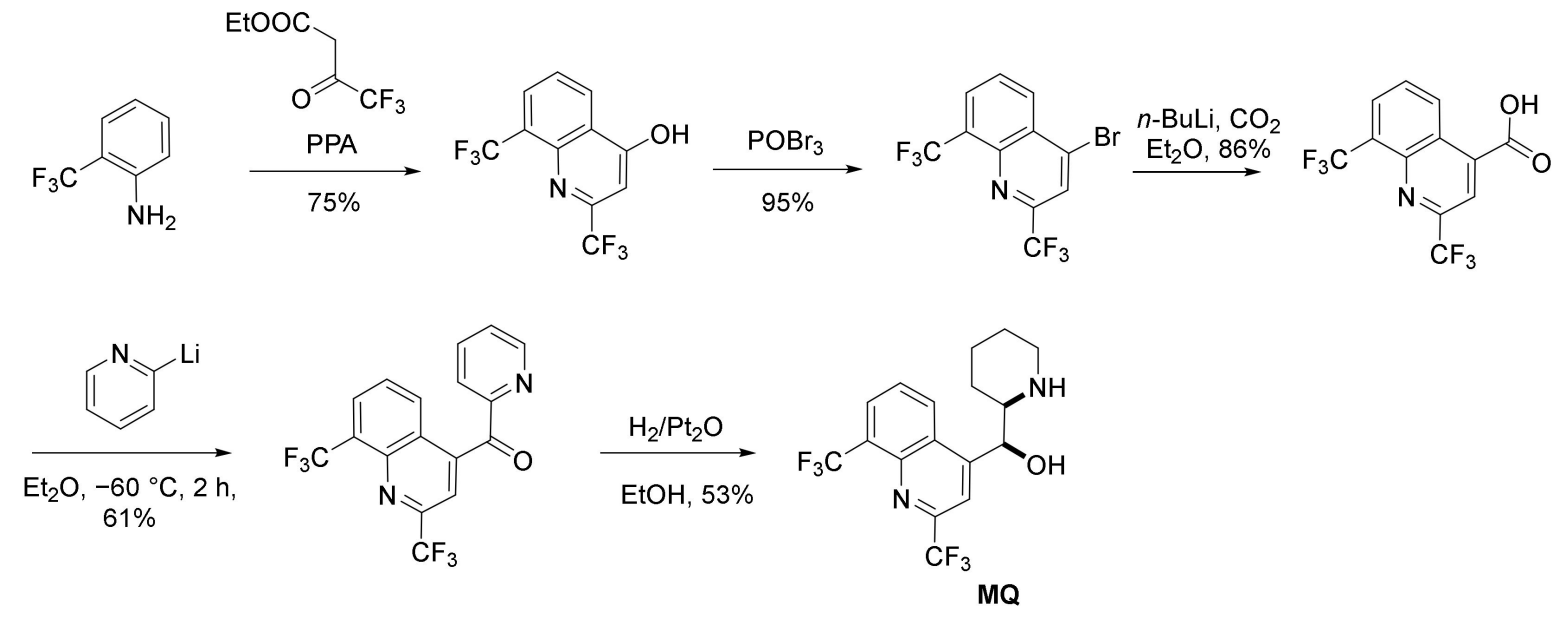
The first synthesis of mefloquine [15].

Mefloquine of *erythro* configuration was first synthesized in the 1970s (Scheme 1) and soon after sold as a drug (e.g., Lariam; racemic mefloquine hydrochloride). Lutz provided the first record on the synthesis of mefloquine in 1971 [15]. Later works included Wittig rearrangement [16], oxidative decyanation [17], and reactions of sulfoxide with Grignard reagent [18]. Mefloquine can be purchased from specialty chemical suppliers at a price of ca. 20 US$/g.

In 1974, Carroll and Blackwell presented the separation of *erythro*-mefloquine enantiomers via 3-bromo-8-camphorsulfonic acid salt [19]. The resolution of the racemic mixture was also conducted with *O,O*′-di-*p*-toluoyl-tartaric acid [20]. The recycling of the material and the use of either enantiomer of the resolving agent provided both enantiomers in a very high yield (86%) and enantiomeric excess (ee above 99%) [21].

The first enantioselective synthesis of (−)-*erythro*-mefloquine via the asymmetric rhodium-catalyzed hydrogenation was reported in 1993 [22,23]. Other enantioselective methods include: asymmetric aldol reaction and the Beckmann rearrangement [24], asymmetric Darzens reaction [25], Pd-catalyzed asymmetric borylative isomerization [26], domino Sonogashira-6π-electrocyclization [27], synthesis from chiral pipecolic acid, enantioselective transfer hydrogenation with ruthenium catalysts [28,29], and the Sharpless asymmetric dihydroxylation [30,31].

Each of the four stereoisomers of mefloquine exhibit slightly different biological properties. For instance, (−)-*threo* and (−)-*erythro*-mefloquine are up to two times less active against malaria parasites. Moreover, the latter is more toxic than its (+) antipode. It is more likely to cause psychotic behavior, and it remains in the organism for up to 2.5 times longer [26]. Nevertheless, ventures for enantiomerically pure mefloquine have not been commercially successful.

Mefloquine acts as an antimalarial in the protozoan cytoplasm and targets the *Plasmodium falciparum* 80S ribosome. It was proven that (+)-*erythro*-mefloquine interacts with residues of PfuL13 such as Leu59 and Glu55 [32]. Mefloquine is slowly metabolized in the liver by cytochrome P450 3A4 (half-life: 3 weeks). The main product of metabolism is carboxymefloquine, which might cause drug interactions [33].


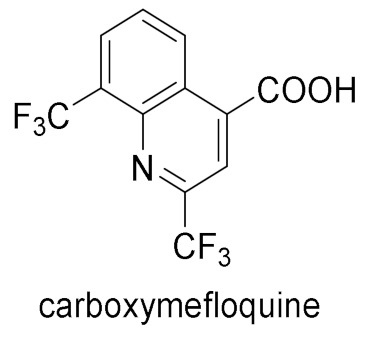


**Figure 2:**
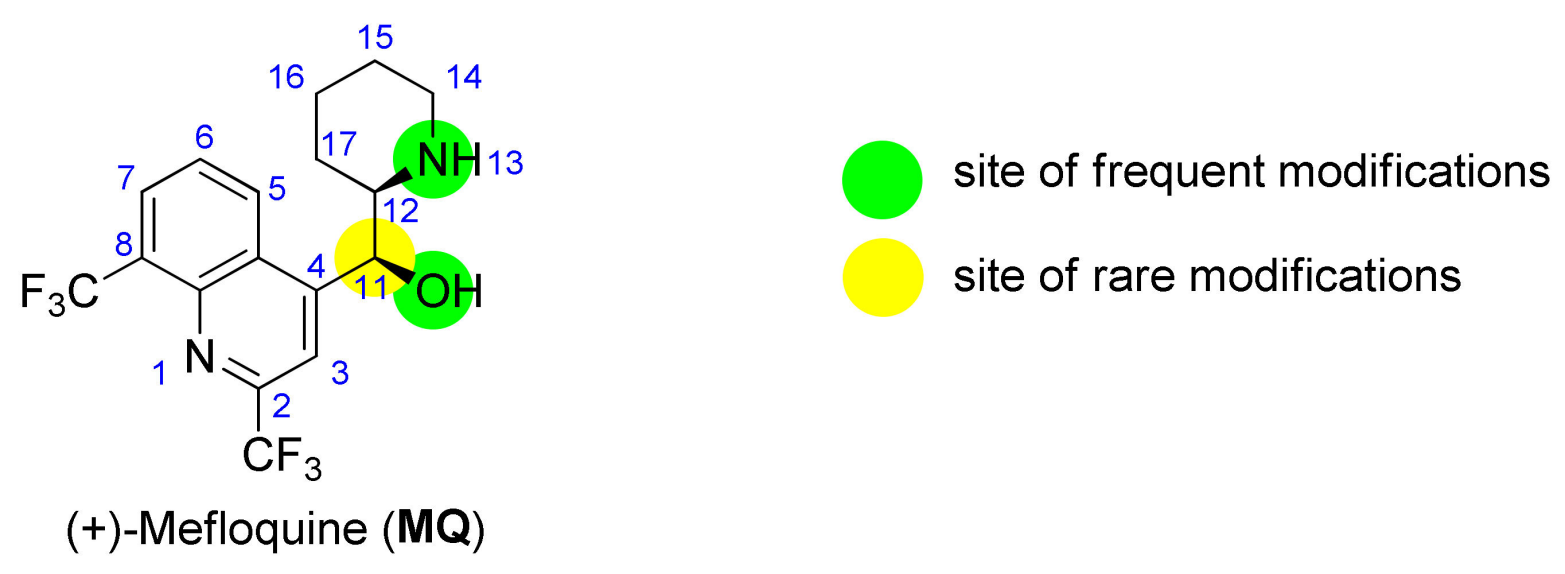
Structure, modification sites, and traditional atom numbering of mefloquine.

Researchers have strived to enhance the biological activity of mefloquine and circumvent the development of resistance to the drug by modifying mefloquine scaffold, sometimes using late-stage modification rather than synthesizing new analogs de novo. Mefloquine possesses two sites, which have been subject to reactions: position 11 (secondary alcohol), and piperidine nitrogen atom at position 13 (Figure 2). To our knowledge, no reactions at other sites including the quinoline ring have been reported in the literature.

### 2.1. Position 13

#### 2.1.1. Amides

Mefloquine acetamide **MQ-1** was produced by either one-step mono-acetylation by acetic anhydride in isopropanol at room temperature or via selective hydrolysis of diacetyl derivative **MQ-2** with LiOH in methanol [19,21].


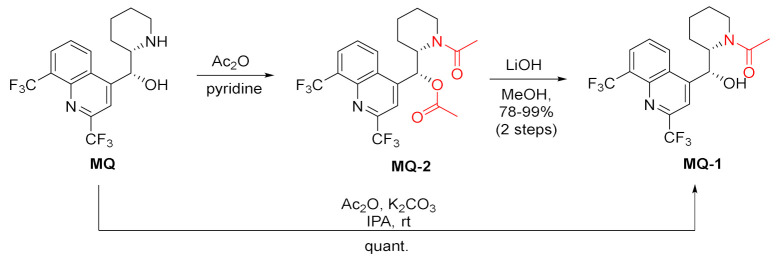


The amide of succinic acid **MQ-5** was obtained effectively using a three-step approach. To assure selectivity, the authors chose trimethylsilyl (TMS) ether protection for the 11-hydroxy group. The acylation with methyl succinyl chloride in the presence of pyridine gave the corresponding amide **MQ-4** an excellent yield. Finally, removal of the protecting group was done with fluoride (TBAF) [34].


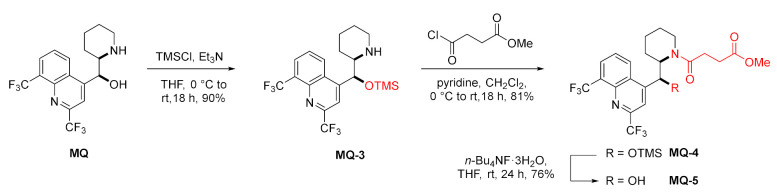


(+)-*erythro*-Mefloquine was acylated with Mosher’s acid using α-methoxy-α-(trifluoromethyl)phenylacetyl chloride (MTPA-Cl) in the presence of *N*,*N*-diisopropylethylamine (DIPEA). Reactions with both enantiomers of MTPA-Cl were performed, but only (*R*)-MTPA-Cl reacted quantitatively. The absolute configuration of (+)-*erythro*-mefloquine hydrochloride was determined as (11*S*,12*R*) by single-crystal X-ray. The synthesis starting from the racemic *erythro*-mefloquine yielded two amide diastereoisomers for each enantiomer of MTPA-Cl. The products presented differences in *R*_f_ values and were successfully separated [35].


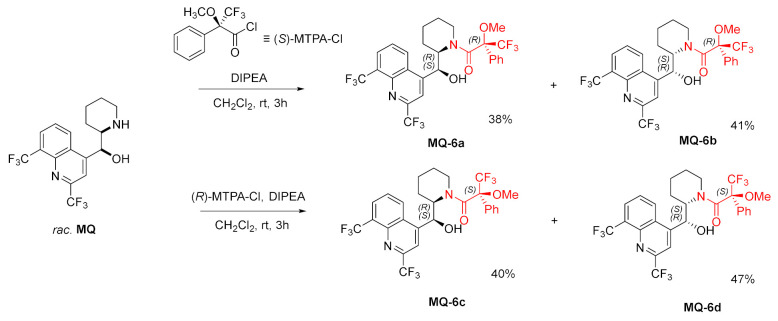


Both (+)-*erythro* and (−)-*threo*-mefloquine were treated with (*S*)-(+)-mandelic acid *t*-butyldimethylsilyl ether activated by uranium salt (1-[bis(dimethylamino)methylene]-1*H*-1,2,3-triazolo [4,5-*b*]pyridinium hexafluorophosphate *N*-oxide, HATU) in the presence of DIPEA. The X-ray structures of the products **MQ-7** and **MQ-9** were used to prove the absolute stereochemistry of (+)-(11*S*,12*R*)-*erythro*-mefloquine and (−)-(11*R*,12*R*)-*threo*-mefloquine [29,30].


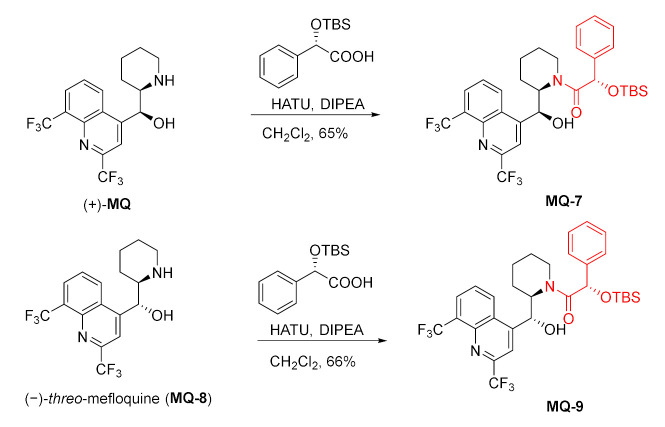


Racemic mefloquine was subjected to the kinetic resolution using polymer reagent for amine resolution (α-PEARL). The use of the chiral active ester of phenylpropionic acid gave corresponding enantioenriched amide **MQ-10**, while the unreacted **MQ** was enantiomerically pure [36].


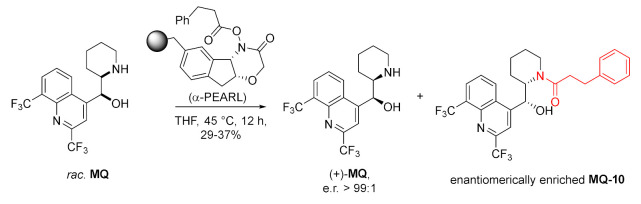


In a parallel kinetic application, two different polymer-supported acylating reagents (pent-4-enoate and *o*-nitrophenylpropionate) with different configurations were applied at once in the reaction with racemic mefloquine. This time a mixture of enantiomerically pure amides **MQ-11** and **MQ-12** was obtained. Both products underwent selective deacylation: pent-4-enamide **MQ-11** with iodine, while *o*-nitrophenylpropionamide **MQ-12** with reduction of the nitro group, followed by mild acidic treatment resulting in intramolecular lactonization. The sequential process allowed for the separation of mefloquine enantiomers without the loss of enantiomeric purity [37].


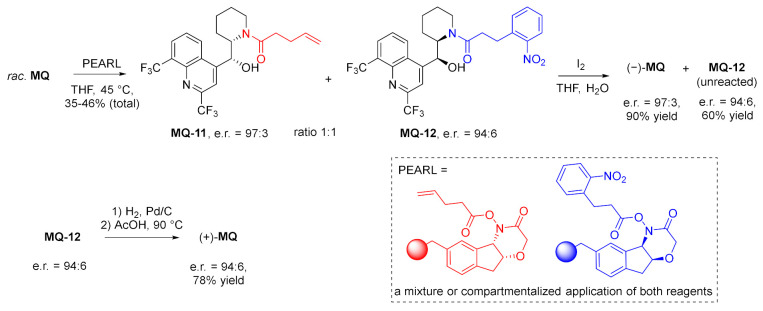


#### 2.1.2. Urethane

Since mefloquine possesses two reactive sites, the use of protective groups was planned to ensure chemoselectivity. For example, the *tert*-butyloxycarbonyl (Boc) protecting group was introduced at the nitrogen 13 atom in mefloquine. The deprotection can be conducted using TFA in CH_2_Cl_2_ [34].


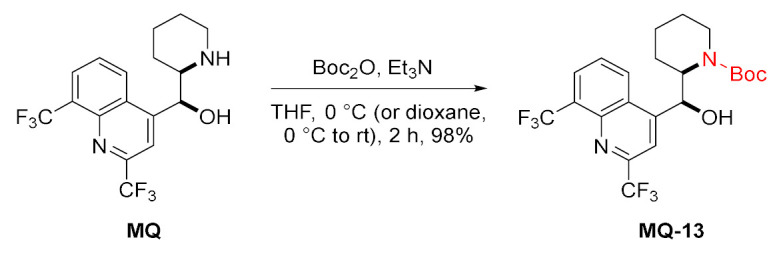


Polycyclic peroxide carbamate **MQ-14a** was formed from mefloquine hydrochloride and functionalized nitrophenyl carbonate (racemate, prepared from 3-hydroxycyclohexanone in a four-step sequence) in the presence of 4-(*N*,*N*-dimethylamino)pyridine (DMAP) and DIPEA. The product **MQ-14a** was obtained as an inseparable mixture of four diastereoisomers in a fair yield. Trioxolane-mediated drug delivery was devised to alleviate the toxicity of mefloquine. It was proven that due to the low brain penetration of the 3″-trioxolane conjugate, decreased brain concentrations of mefloquine were found in mice treated with **MQ-14a** than in the animals on the unmodified drug. It was assumed that when *Plasmodium* digested hemoglobin and heme was produced, iron(II) induced the cleavage of the endoperoxide bond in the conjugate by means of a Fenton-like reaction. This affords a ketone, which releases mefloquine after decarboxylation. The antimalarial activity of the conjugate **MQ-14a** against *P. falciparum* W2 strain was similar to the parent compound and arterolane (EC_50_ 2.5–3.0 nM) [38].


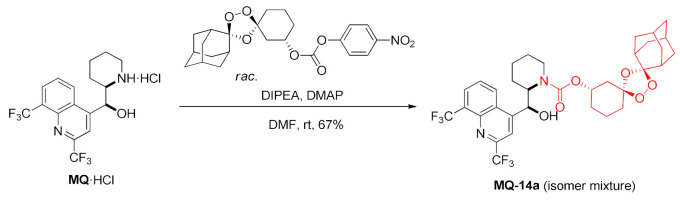


More carbamates (**MQ-14b**−**d**) were prepared from sterically shielded trioxolanes. Their activity (IC_50_: 24–74 nM) against *P. falciparum* W2 was higher than for chloroquine, yet lower than for artefenomel (IC_50_: 285 and 9.0 nM, correspondingly) [39].


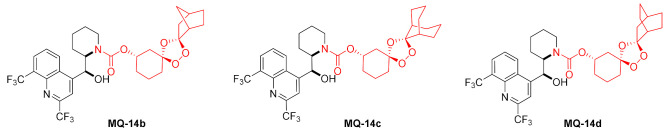


##### 2.1.3. (thio)Urea

4-Chlorophenyl isocyanate reacted with mefloquine in the presence of a base (Et_3_N) to form the corresponding urea **MQ-15** in a fair yield. The product displayed moderate antituberculotic activity [40].


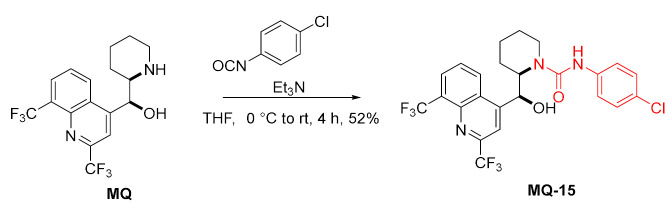


*erythro*-Mefloquine reacted with allyl isothiocyanate to form 13-*N*-allylthiourea derivative **MQ-16** in a very good yield. The X-ray crystal structure of this mefloquine derivative provided proof for the absolute stereochemistry directly based on anomalous scattering [41].


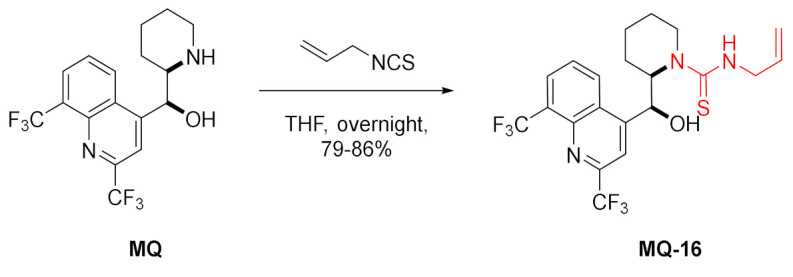


#### 2.1.4. Alkyl Derivatives

A series of *N*-substituted mefloquine derivatives was prepared using alkyl bromides, in the presence of a base (K_2_CO_3_ or Et_3_N) in poor to moderate yields. The mefloquine-derived tertiary amines **MQ-17**−**23** were investigated as potential anti-tuberculotic (anti-TB) agents. It transpired that the presence of the benzyl group at the N-13 atom in mefloquine increased the activity of **MQ-20** against *Mycobacterium tuberculosis* H_37_Rv in respect to (+)-*erythro*-mefloquine hydrochloride with MIC: 6.7 µM in MABA (microplate alamarBlue assay) and 7.3 µM in LORA (low oxygen recovery assay). Additionally, the benzyl derivative **MQ-20** exhibited low cytotoxicity. However, the 4-pyridylmethyl compound **MQ-22** was approximately 70% less active than *erythro-N-*benzylmefloquine **MQ-20**. Polar electron-withdrawing substituents in the *para* position of the benzyl group (sulfonamido or carboxyl groups) also decreased the anti-TB activity. No activity was observed for compounds with acetic acid **MQ-17** and acetamide residues **MQ-18** as substituents. In the same assay, the urea **MQ-15** and 13-Boc-mefloquine **MQ-13** showed moderate activity [40].


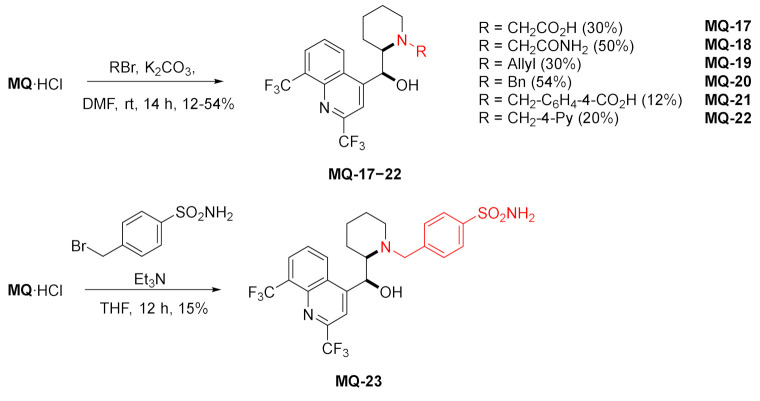


A conjugate of artemisinin, an established antimalarial drug, and mefloquine was prepared. The piperidinyl amino group (N-13) of mefloquine was alkylated using allyl bromide derived from 10-CF_3_-artemisinin in the presence of triethylamine resulting in the formation of the tertiary amine **MQ-24** in a moderate yield. Under these conditions, the hydroxy group is unaffected. The link between both drugs in **MQ-24** is not hydrolyzable [34].


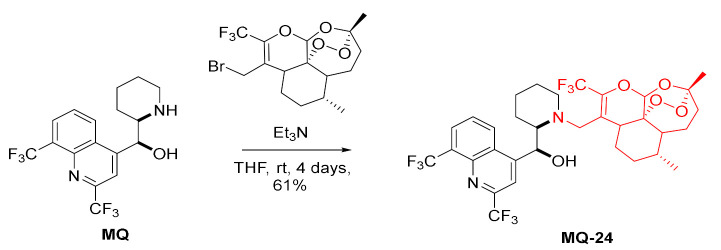


*erythro*-13-Methylmefloquine was prepared in the Eschweiler–Clarke reaction using formic acid and formaldehyde. The tertiary amine **MQ-25** was further converted to the *N*-oxide **MQ-26** by the oxidation with *m*-chloroperoxybenzoic acid. Both products were obtained in a fair yield. Unlike *erythro*-mefloquine, compounds **MQ-25** and **MQ-26** exhibited no contractual effect on the isolated mouse diaphragm and no inhibition of directly simulated twitch response [42].


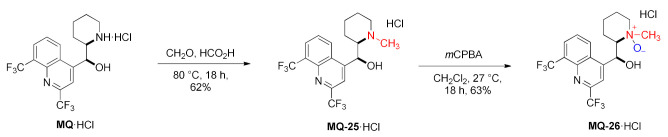


In a similar approach, the cyanoethyl alkylated product **MQ-27** was obtained in the Michael addition. The corresponding reaction of mefloquine and acrylonitrile gave a very good yield. Next, oxidation with *m*-chloroperoxybenzoic acid afforded hydroxylamine **MQ-28**. Hypervalent iodine(III) reagents are capable of trifluoromethylating *N*,*N*-dialkylhydroxylamines. Here, the reaction of **MQ-28** with Togni reagent accomplished trifluoromethylation at the *N*-hydroxy group affording the ether **MQ-29**. In order to improve chemoselectivity, the authors decided to both activate the hypervalent iodine reagent with a Lewis acid and deprotonate **MQ-28** with an adequate base, therefore trimethylsilyl triflate (TMSOTf) and tetramethylguanidine (TMG) were used. The mechanism involved nitroxyl and CF_3_ radicals. The native 11-hydroxy group in mefloquine did not react under these conditions [43].


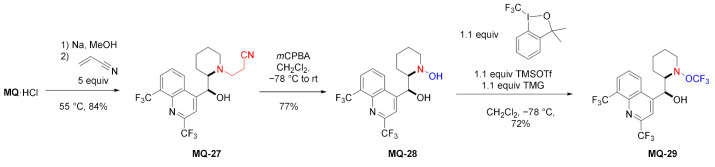


Mefloquine underwent stereospecific strain-release cyclopentylation with chiral 1-sulfonyl-bicyclo [2.1.0]pentane (*housane*) acting as a covalent reactive group. The reaction yielded a disubstituted cyclopentane derivative **MQ-30** and a complete stereotransfer was observed. The difference in reactivity of racemic and enantiopure sulfone reagent was negligible (35 and 32% yield, respectively). The secondary amino group reacted chemoselectively and the hydroxy group remained unaffected [44].


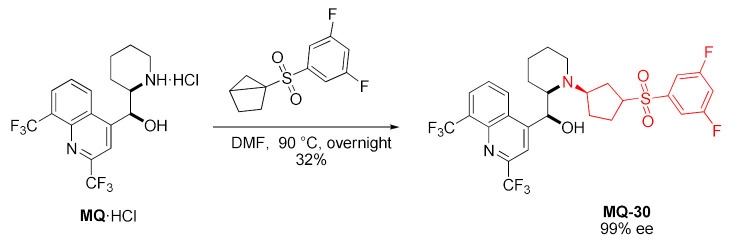


### 2.2. Position C-11

#### 2.2.1. 11-Inversion

*erythro*-Mefloquine can be converted to the *threo* isomer **MQ-8** in a three to fourstep process. (+)-(11*R*,12*S*)-*N*-acetyl mefloquine (**MQ-1**) under treatment with thionyl chloride undergoes rearrangement via a probable oxazolinium species to the *O*-acetyl derivative **MQ-31** with inversion of configuration at position 11. Acid hydrolysis affords (+)-(11*S*,12*S*)-*threo*-mefloquine hydrochloride **MQ-8**·HCl in an excellent overall yield. Tests on mice showed that neither isomer exhibited substantial differences in activity against *P. berghei* [19].


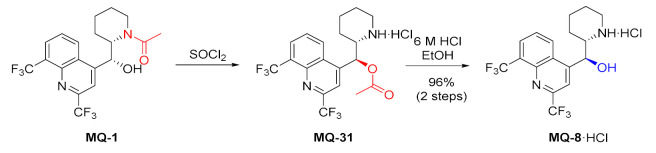


#### 2.2.2. 11-Ketone

Rutjes and Vlieg presented a method for the deracemization of *erythro*-mefloquine. First, the Boc-protecting group at the N-13 atom was introduced. Then, the secondary alcohol was converted to the corresponding ketone **MQ-32** in the Dess–Martin oxidation. In the process, one stereogenic center at position 11 was completely lost, while the remaining stereogenic center was labile due to tautomerization. Strong acids such as HCl and a vast number of sulfonic acids were screened for the removal of Boc and crystallization of salts in the form of a racemic conglomerate. Out of more than 30 crystal structures, only **MQ-33**·di(biphenylsulfonate) dihydrate formed the racemic conglomerate and allowed for the resolution of enantiomers using Viedma ripening. Eventually, the resolved ketone **MQ-33** was reduced with NaBH_4_ to the (+)-(11*S*,12*R*)-*erythro*-mefloquine. The overall yield was 83% [45].


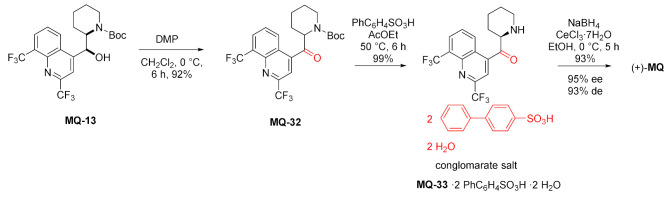


#### 2.2.3. Photochemical Degradation

Generally, mefloquine exhibits low phototoxicity. Photodegradation occurs more effectively at higher pH. Carbon 11-centered benzyl type radical was postulated as a precursor for all the observed degradation products. Subsequent reactions lead to the cleavage of adjacent bonds. A number of products have been identified after irradiation in water [46,47]. In methanol, photodegradation essentially leads to two defined products arising from the cleavage of the C-11–C-12 bond, mostly carboxymefloquine methyl ester (**MQ-34a**, 55% yield) and a small quantity of corresponding benzyl alcohol **MQ-34b** [48].


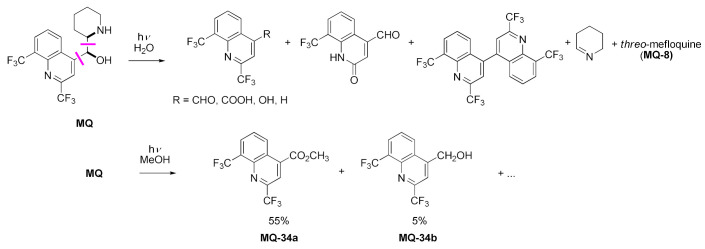


### 2.3. Position O-11

Despite the wide reactivity of *Cinchona* alkaloids at their central hydroxy group, much less chemistry has been performed solely with the hydroxy group of mefloquine. Only the TMS group has been introduced selectively (*vide supra*) [34]. All the remaining cases resort to the use of a protecting group at the nitrogen 13 atom to achieve selective reactivity of the hydroxy group.

#### 2.3.1. Esters

Boc-protected mefloquine (**MQ-13**) reacted with succinic and glutaric anhydrides to obtain ester acids homologs **MQ-35** and **MQ-36**. Then, carboxylic acid groups were transiently converted to acyl chlorides with thionyl chloride and used to acylate hydroxy group of atovaquone (ATQ), another antimalarial drug. The overall yields were rather poor. During the process, the Boc group was partially removed from the succinic derivative, giving a mixture of the protected (**MQ-37**, 21% yield) and the deprotected product (**MQ-38**, 11% yield). Treatment of **MQ-37** with methanolic HCl removes Boc in an excellent yield. The esterification process of homologous **MQ-36** resulted only in the deprotected product **MQ-39**. Both the derivatives **MQ-38** and **MQ-39** turned out to be more active (IC_50_: 0.6–1.1 nM) against *P. falciparum* F32-TEM than the unmodified drugs **MQ** and atovaquone (IC_50_: 87 and 2 nM) [49].


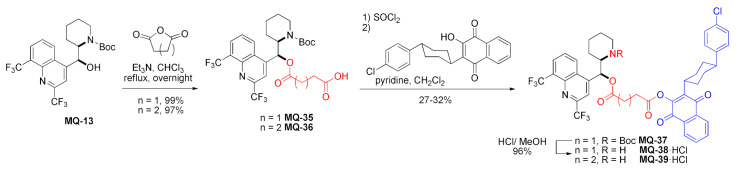


In a preceding approach, mefloquine was linked to artemisinin using an ester linker. The reaction of ester carboxylic acid **MQ-35** with hydroxyether artemisinin derivative was performed under carbodiimide activation in the presence of DMAP. The corresponding product **MQ-40** was isolated in a good yield. Finally, removal of the Boc-protecting group with TFA afforded the drug conjugate **MQ-41** in only a fair yield. Under these conditions, some transfer of acyl group from oxygen 11 to nitrogen 13 atom occurred, resulting in amidoester by-product **MQ-42** [34]. This reaction did not occur in the synthesis of **MQ-38** and **MQ-39** [49].


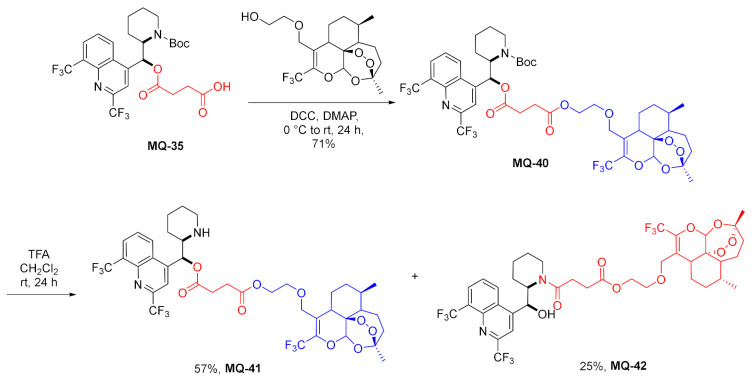


The ester linkage was devised to bond together CF_3_-artemisinin and mefloquine into a hybrid molecule, in which in vivo hydrolysis could release both drugs. Compounds **MQ-41** and **MQ-24** (*vide supra*) were highly effective against *P. falciparum* (strains: F32, Thai, FcB1, and K1). It is noteworthy that the ester **MQ-41** performed better than the permanently linked tertiary amine **MQ-24** (IC_50_: 2.4–6 vs. 10–17 nM, respectively) as well as unmodified mefloquine and chloroquine (IC_50_: 2.8–23 and 14–183 nM, correspondingly). No cross-resistance with **CQ** or **MQ** was observed for **MQ-24**. The authors recognized their products as a part of covalent bi-therapy, which aims to increase the therapeutic effect of a drug and decrease the possibility of drug resistance. The synthesized compounds consist of two biologically active modules. Such an approach has been reported to be effective in the case of synthetic endoperoxide-chloroquine and statine-primaquine derivatives. The mefloquine and 10-trifluoromethyl artemisinin combination improved metabolic stability and increased activity when compared to other artemisinin-based compounds [34].

#### 2.3.2. Carbamates

The use of *Cinchona* alkaloid carbamates in enantioselective separations by Lindner and coworkers resulted in the development of commercial products [50]. In 2001, the same group reported racemic mefloquine-*O*-*tert*-butylcarbamate **MQ-44** obtained in a three-step sequence. The product **MQ-44** along with a few different chiral compounds was tested as a basic selectand for the performance test of (*S*)-3,5-dinitrobenzoyl-leucine chiral selector in nonaqueous capillary electrochromatography (CEC). The experiment proved that the separations relying on stereoselective ion-pair formation between the selector and the selectands proceeded in excellent stereoselectivity [51]. In subsequent works, **MQ-44** was used in performance tests of multiple chiral stationary phases (CSP) in CEC [52,53,54,55,56,57], HPLC [58,59,60,61], and supercritical fluid chromatographic (SFC) separations [62,63]. In all cases, strong ion exchangers were needed to achieve good separations.


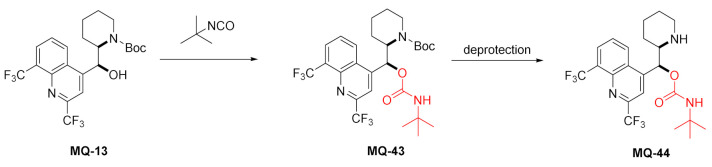


The works by Lindner also mention enantioseparations of some 13-alkyl mefloquine derivatives (*N*-allyl **MQ-19**, *N*-methyl **MQ-25**, *N*-undecen-1-yl-mefloquine) and *N*-allyl-*O*-*tert*-butylcarbamoylmefloquine **MQ-46** [62,64]. The enantiomerically pure allyl derivative **MQ-19** was obtained by resolution of diastereomeric *O*,*O*-diacetyl-l-tartaric acid esters **MQ-45**. The reaction of **MQ-19** with *tert*-butyl isocyanate catalyzed by dibutyltin laurate gave the corresponding carbamate **MQ-46** in a very good yield [64].


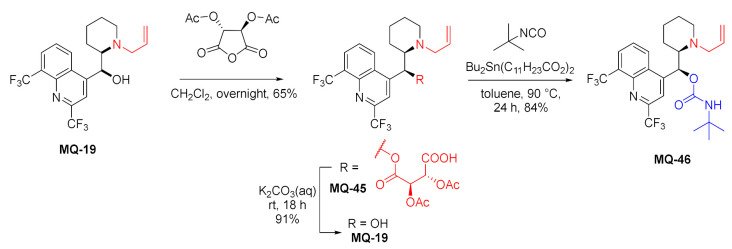


### 2.4. Modifications Involving Both Positions 11 and 13

*erythro*-Mefloquine was methylated at two positions at once when deprotonated with sodium hydride and reacted with iodomethane. The transformation resulted in the tertiary amine ether **MQ-47** in a moderate yield [65].


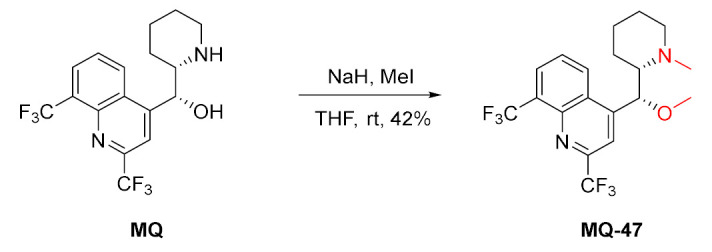


Acetylation under forceful conditions with acetyl anhydride at above 90 °C resulted in the diacetyl product **MQ-2** (*vide supra*) [66]. Similarly, fatty acid chlorides were used to N,O-diacylate mefloquine. Reactions with palmitoyl and oleoyl chlorides in the presence of catalytic DMAP resulted in very good yields of products **MQ-48** and **MQ-49**. These products were characterized by increased lipophilicity, three-times better solubility in oil, and were, therefore, suitable for use in lipid emulsions. Subsequently, the products were contained in 20% sesame oil-in-water submicron emulsions, which were physically stable. However, their antimalarial activity was completely lost [67].


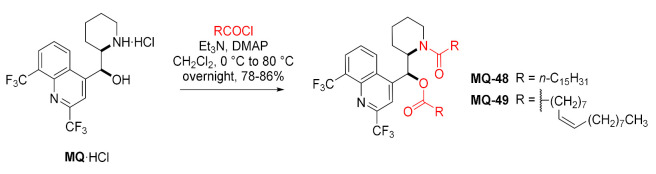


#### 2.4.1. Heterocyclic Derivatives: Aziridine Ring and Ring-Opening Reactions

*erythro*-Mefloquine, under the Appel reaction conditions, undergoes dehydration to form aziridine **MQ-50** with inversion of configuration (*threo*) as shown by Rösner and Brossi. The reaction was carried out with mefloquine free base, triphenylphosphine, triethylamine, and carbon tetrachloride in acetonitrile. The product **MQ-50** was obtained in a fair yield and its structure was confirmed with X-ray. In the solid phase, the aziridine **MQ-50** displays photochromic properties: Upon irradiation of the colorless product with UV light (366 nm), it changes color to purple, which disappears more slowly when kept in the dark. Nonetheless, the process does not induce decomposition of the compound. The aziridine **MQ-50** reacts with acetic anhydride at room temperature to form the *erythro*-*N*,*O*-diacetyl derivative **MQ-2**, identical to that obtained by reacting *erythro*-mefloquine with acetic anhydride at 90 °C. In this sequence of reactions, configuration of the initial mefloquine is retained [66]. Attempts to obtain the *erythro*-diastereoisomer of aziridine were unsuccessful.


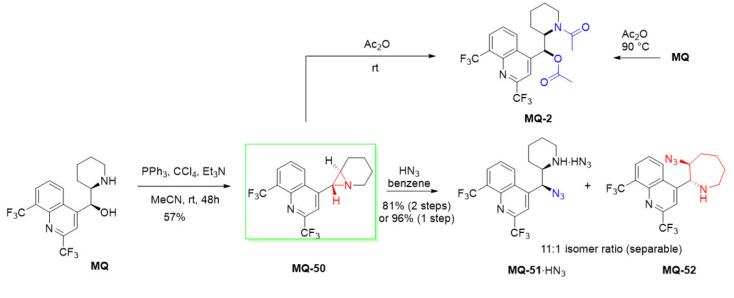


Ring-opening of the aziridine **MQ-50** with hydrazoic acid produced the corresponding azide **MQ-51** very efficiently. In addition, a small contribution of ring-opening at the alternative site resulted in the isolation of azide with azepane ring **MQ-52**. The yield of azides from the pure aziridine was nearly quantitative, while a two-step process without proper purification of the aziridine **MQ-50** resulted in 81% yield. This suggests that the aziridine is formed more efficiently than it is isolated. The aminoazide **MQ-51** was quantitatively hydrogenated to *erythro*-11-aminomefloquine **MQ-53**. The aminoazide **MQ-51** was also efficiently benzylated with benzyl bromide or methylated under the Eschweiler–Clarke conditions. However, too basic conditions in the Schotten–Bauman reaction caused epimerization at position 11. Identical products **MQ-54**–**55** were obtained from the Mitsunobu reaction of *N*-alkyl *erythro*-mefloquines (**MQ-20** and **MQ-25**) with an azide source. Retention of configuration is consistent with the aziridinium ion participation. The azides **MQ-54**–**55** were hydrogenated to obtain the corresponding amines **MQ-56**–**57** [21].


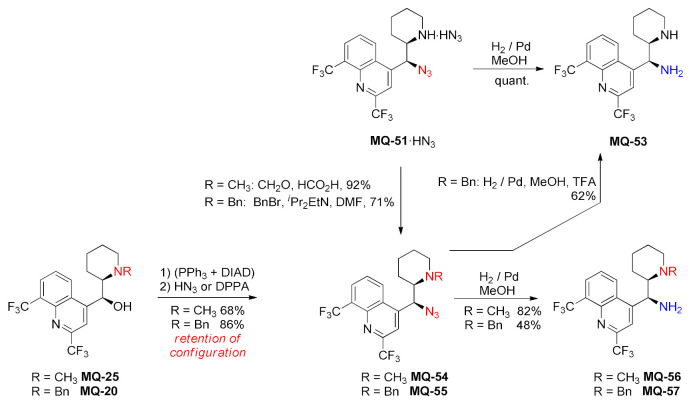


Consequently, the *threo* isomer of 13-benzyl mefloquine (**MQ-58**) gave *threo*-azide **MQ-59**. The formed derivative was hydrogenated to afford the primary-tertiary amine **MQ-60,** which is reminiscent of *epi*-9-aminocinchona alkaloids. Removal of the benzyl group was performed by hydrogenation in acidic solutions [21].


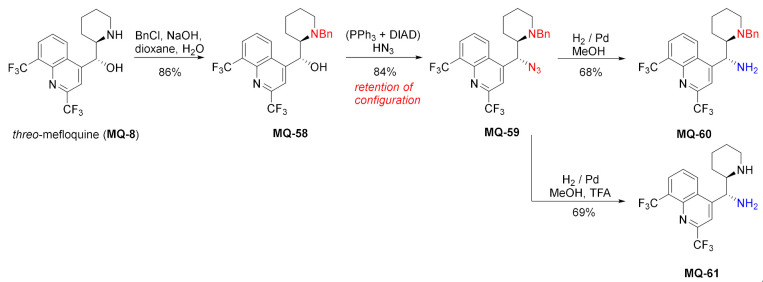


Since the replacement of the hydroxy group for a primary amino group in quinine afforded a potent asymmetric organocatalyst, the aminomefloquine derivatives were assayed for the catalytic activity. Indeed, (+)-*erythro*-11-aminomefloquine **MQ-53** was proven as a better catalyst than 9-*epi*-aminoquinine in the asymmetric Michael Addition of nitromethane to cyclohexanone in terms of enantioselectivity, delivering the catalytic product with a very good ee (93%) and a moderate yield [21].

#### 2.4.2. Imidazolidine Ring

Mefloquine derivatives with a fused imidazolidinone ring were prepared in the reaction of 11-aminomefloquines **MQ-53** and **MQ-61** with 1,1′-carbonyldiimidazole (CDI) or phosgene in moderate yields. The products **MQ-62**−**63** were obtained to assist with the assignment of the relative configuration of the initial 11-aminomefloquines [21].


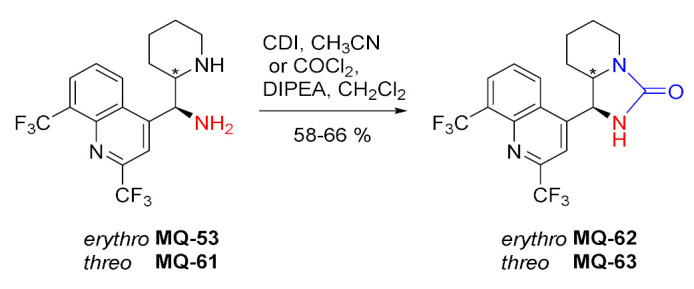


#### 2.4.3. Oxazolidine Ring

A library of products with a fused oxazolidine ring were obtained by treating mefloquine with various carbonyl compounds. The reaction was performed mostly with benzaldehyde derivatives in refluxing toluene, while liberated water was azeotropically removed. In the product, a new stereogenic center is formed at the oxazolidine carbon atom 2, and due to the fusion of oxazolidine and piperidine rings, the molecules are more rigid, which generates an additional stereogenic center at the piperidinyl nitrogen atom [68]. The reaction of mefloquine and acetone in the presence of the acidic catalyst resulted in a fusion of 2,2-dimethyl-oxazolidine ring, confirmed by X-ray [69]. The products **MQ-64a**-**q** (stereoisomer mixtures) were tested in the anti-TB assay (against *M. tuberculosis* ATCC 27294). A wide variety of substituents provided information on the structure–activity relationship (SAR) for the introduced aromatic ring. It turned out that electron-donating groups such as -OH and -OMe increase the anti-TB potential [68]. Further development indicated that derivatives with a 2-methoxyphenyl **MQ-64f**, 2,3-dimethoxyphenyl **MQ-64h**, and 5-nitrothien-2-yl **MQ-64o** substituents (MIC ca. 12 µM) were more active than mefloquine, and their activity was similar to ethambutol (MIC = 33 and 16 µM, respectively). No cytotoxicity to normal cells was observed [68,70]. A similar set of compounds was tested for antiproliferative activity. It was determined that derivatives with electron-rich aryl and heteroaryl substituents were more cytotoxic against cancer cells such as ovarium cancer, leukemia, and glioblastoma cells compared to mefloquine [71].


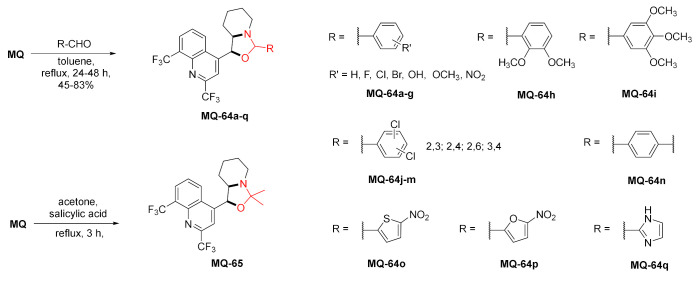


Another set of derivatives obtained from *erythro-* and *threo*-mefloquine was described in a patent as a part of the human adenosine A_2A_ receptor antagonists. The cyclic products **MQ-66a**–**b** were obtained by treating *erythro*-mefloquine with formaldehyde to give oxazolidines in fair to good yields. The reaction with CDI gave oxazolidinones **MQ-67a**–**b** in a rather poor yield [65].


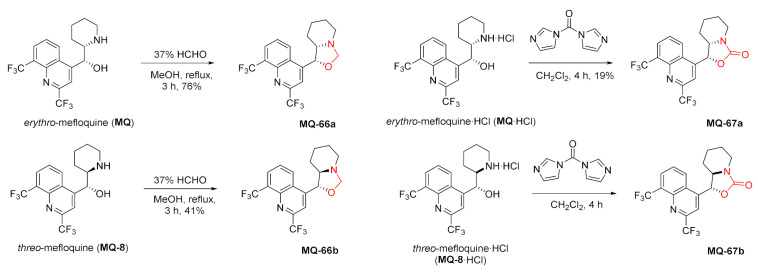


A similar reaction was reported in the literature to give the sulfur analo **MQ-68**. Fused oxazolidinethione ring was obtained in the reaction of mefloquine with 1,1′-thiocarbonyldiimidazole (TCDI) in the presence of triethylamine. Again, the reaction resulted in a rather poor yield. The product did not display anticipated anti-TB activity [40].


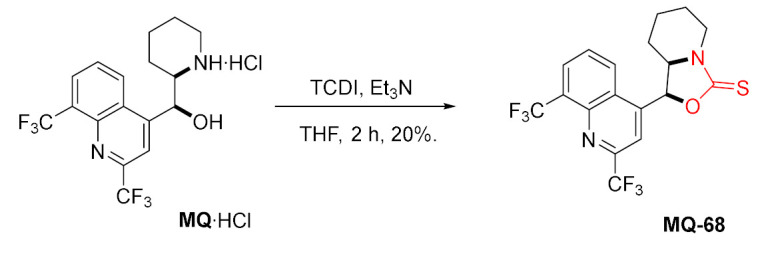


#### 2.4.4. Oxazaborolidine Ring

Wardell et al. prepared solvates of racemic *erythro*-mefloquinium tetraphenylborate, which upon heating above their melting points gave mefloquine-*N*,*O*-diorganoborinate ester **MQ-69**. The compound is characterized by hydrolytic stability due to the intramolecular N–B coordination bond. The alternative single-step process in solution turned out to be less effective. On the other hand, the attempted reaction with sodium tetraethylborate failed due to decomposition. The MIC values from in vitro assays against *M. tuberculosis* for mefloquinium tetraphenylborate and the mefloquine oxazaborolidine derivative **MQ-69**∙EtOH were 12 and 50 µg/mL, respectively. The former was presented to be slightly more active than mefloquine hydrochloride (25 µg/mL), yet worse than ethambutol or rifampicin (2–3 µg/mL) [72].


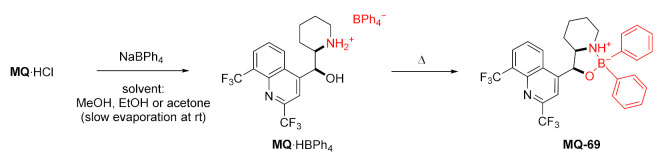


#### 2.4.5. Morpholine Ring

The fusion of a six-membered ring involving mefloquine oxygen 11 and nitrogen 13 atoms was accomplished in acetylation-alkylation with chloroacetyl chloride. The reaction carried under the Schotten–Bauman conditions delivered the corresponding morpholinone **MQ-70**. The subsequent reduction with borane afforded the morpholine derivative **MQ-71**. Both steps were conducted in rather poor yields [65].


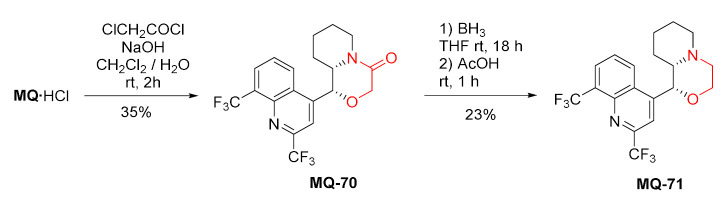


### 2.5. Metal Complexes

**Figure 3:**
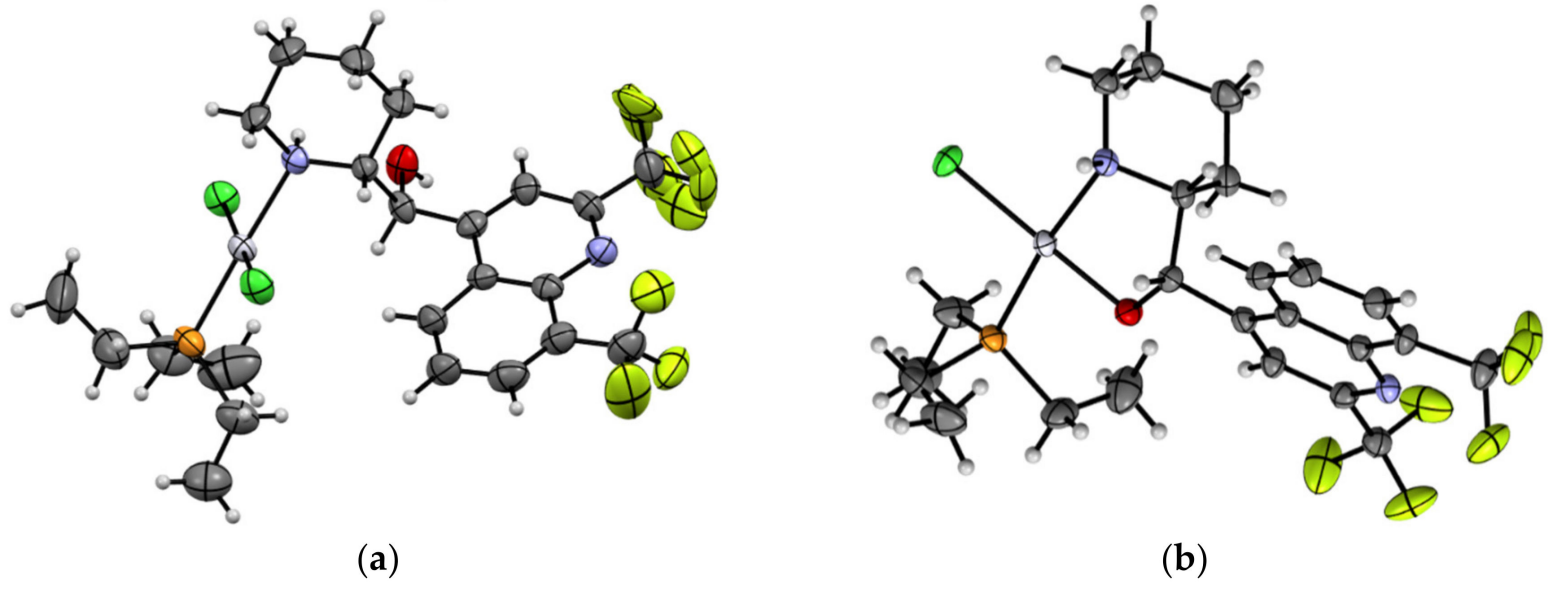
X-ray structures of chloro(triethylphosphine)platinum(II) complexes with **MQ** as mono (**a**) and bidentate ligand (**b**) [73].

From the coordination chemistry perspective, mefloquine possesses three metal binding sites: the nitrogen atoms at position 13 (piperidine) and 1 (quinoline), and the oxygen 11 atom (hydroxy group). The formation of complexes with palladium(II) and platinum(II) was studied. Under neutral conditions, both metal ions formed monodentate complexes with the piperidine nitrogen atom. Deprotonation of the hydroxy group, either spontaneous (3 days reaction time) or caused by external base, resulted in bidentate N,O-complexes (Figure 3). Protonated mefloquine species formed a coordination bond only with platinum(II). Based on spectroscopic experiments, it was postulated that the quinoline nitrogen 1 atom was involved [73].

**Figure 4:**
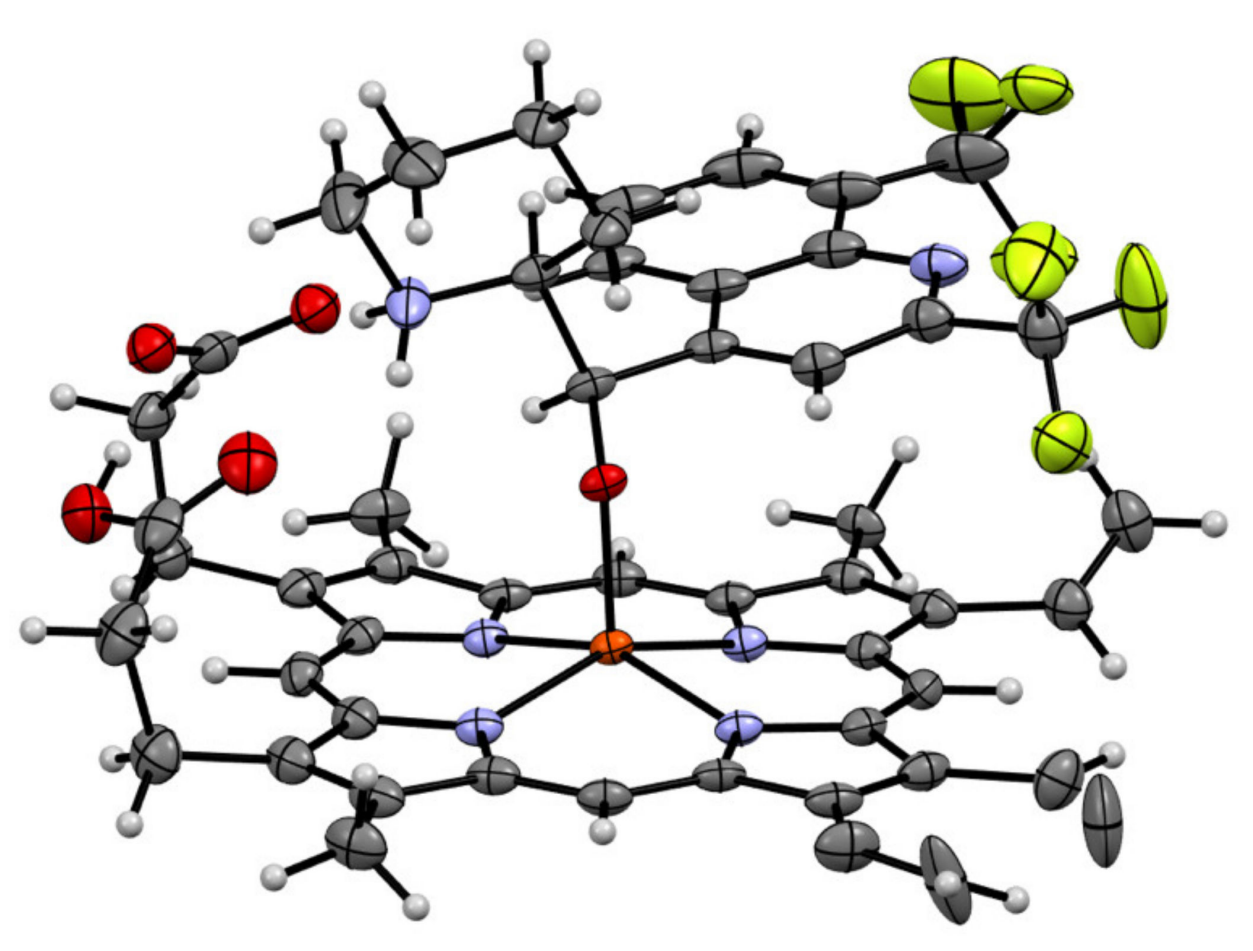
Partial view of X-ray structure of mefloquine complex with Fe(III) protoporphyrin IX [76].

*Plasmodium* species feed on hemoglobin in erythrocytes. In the process, heme is formed, which is toxic for the pathogen. In order to remove it, the parasite metabolizes it to α-hematin and eventually to hemozoin (β-hematin) by means of biocrystallization [74]. This process is inhibited by quinine, which forms a coordination bond with protoporphyrin IX [75]. A similar study was performed with mefloquine. Binding constants were similar for **MQ** and quinine or quinidine, however, under the studied conditions, no coordination compound with **CQ** was observed. The adequate crystalline complex of **MQ** was obtained from the acetonitrile solution (Figure 4). The coordination bond with iron involves the deprotonated hydroxy group, similarly to *Cinchona* alkaloids. Additionally, ionization of carboxylic acid and piperidine, as well as π-stacking interactions were observed between the mefloquine and protoporphyrin parts. Minor differences in the activity between (+) and (−)-mefloquine were attributed to different abilities to bond to iron(III) protoporphyrin IX [76].

## 3. (Hydroxy)Chloroquine

**Scheme 2:**
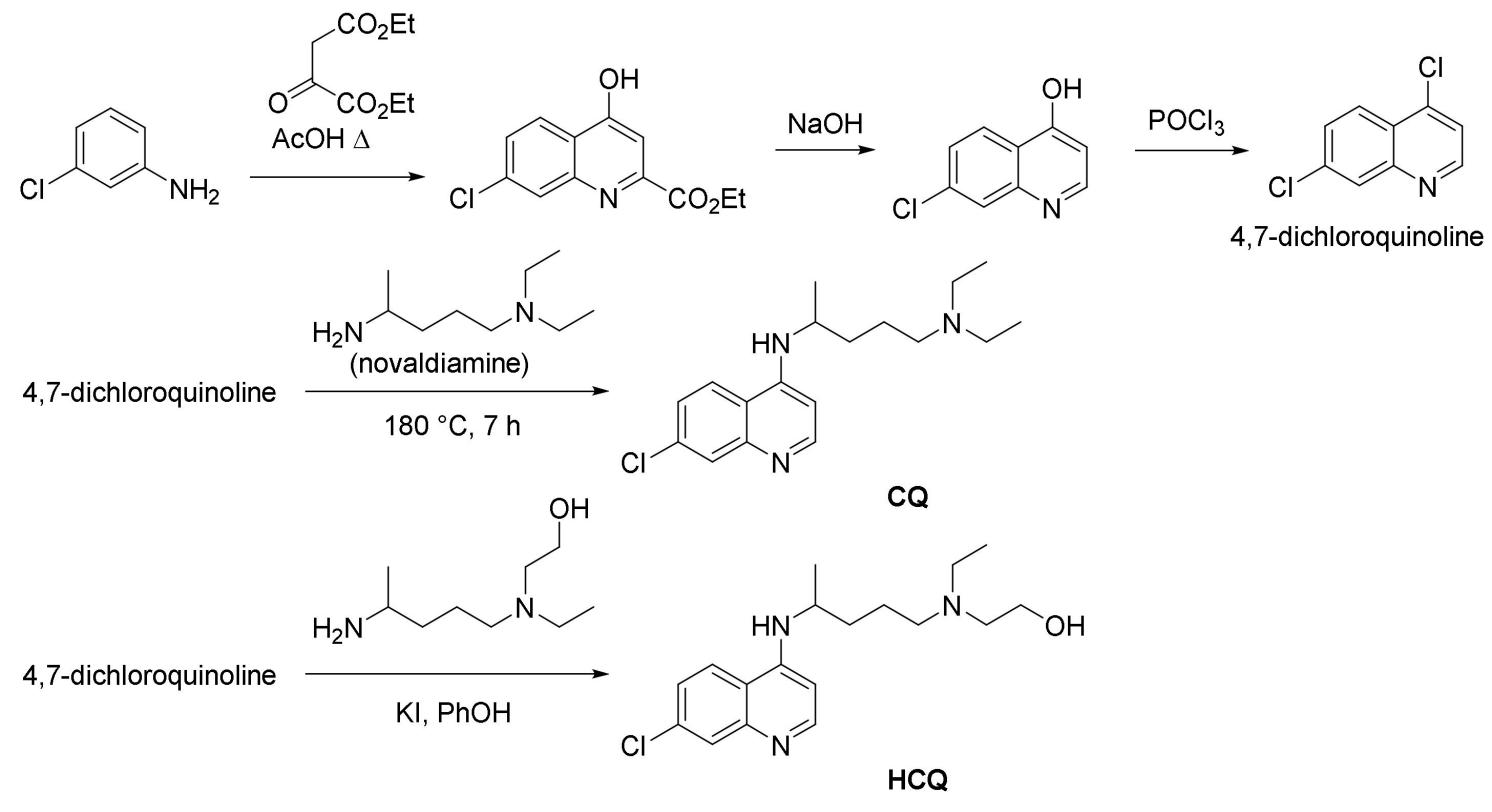
Synthesis of chloroquine and hydroxychloroquine.

Chloroquine (**CQ**) was first synthesized in the 1930s as an antimalarial agent. It is obtained in a convergent synthesis, in which aliphatic (novaldiamine, 2-amino-5-dimethylaminopentane) and quinoline parts (4,7-dichloroquinoline) are combined in the final step of the aromatic nucleophilic displacement. Both reactants are available at a low price (ca. 1 $US/g). Nevertheless, 4,7-dichloroquinoline can be prepared in a three-step sequence. First, Conrad–Limpach condensation between *m*-chloroaniline and diethyl oxalacetate yields 7-chloro-4-hydroxyquinoline-2-carboxylic acid ethyl ester. Basic hydrolysis and decarboxylation afford 7-chloro-4-hydroxyquinoline, in which the hydroxy group is exchanged for chlorine by the action of phosphoryl chloride. Final coupling with adequate primary-tertiary diamine produces chloroquine [77,78] or hydroxychloroquine [79] (Scheme 2).

Hydroxychloroquine (**HCQ**), in which one of the ethyl groups of **CQ** was replaced with the hydroxyethyl group, has been developed as a medicine that is safer and related to fewer side effects [80]. The use of **CQ** and **HCQ** as antimalarials is now limited due to the emerging chloroquine-resistant *P. falciparum* strains. For research purposes, the following strains are often used [81]:

Chloroquine-sensitive: FcB1, PFB, F32, NF-54 (3D7), FC27 (D10), D6, GB4, T9-96, HB3, ITG2F6, GC03, and Thai.

Chloroquine-resistant: K1, FCR3, W2 (W2mef, Dd2) W1a, W1b, FcB1R, E1a, E1b, P1, 7G8, 7G8, and V1 (V1/S).

Both **CQ** and **HCQ** display immunomodulatory effects that have been exploited in the management of a number of autoimmune diseases including lupus (systemic lupus erythematosus, SLE) rheumatoid arthritis, Sjörgen’s syndrome, and antiphospholipid antibody syndrome [82]. Both compounds display antiviral properties [83], however. high hopes for curing COVID-19 proved immaterial [84].

Racemic chloroquine is sold as diphosphate (e.g., Resochin, Aralen) and sulfate (e.g., Nivaquine). Hydroxychloroquine is marketed as sulfate (e.g., Plaquenil). The drugs are absorbed from the intestinal tract [82,85]. Desethylchloroquine is the main metabolite of chloroquine produced by the human hepatic enzymes P450 2C8 and 2D6 [86]. Other products are bisdesethylchloroquine and 7-chloro-4-aminoquinoline [87].


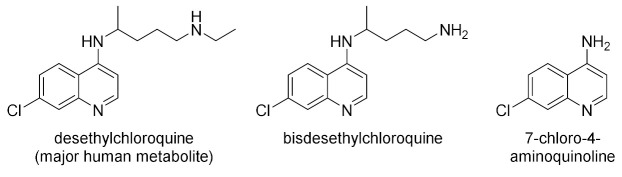


**Figure 5:**
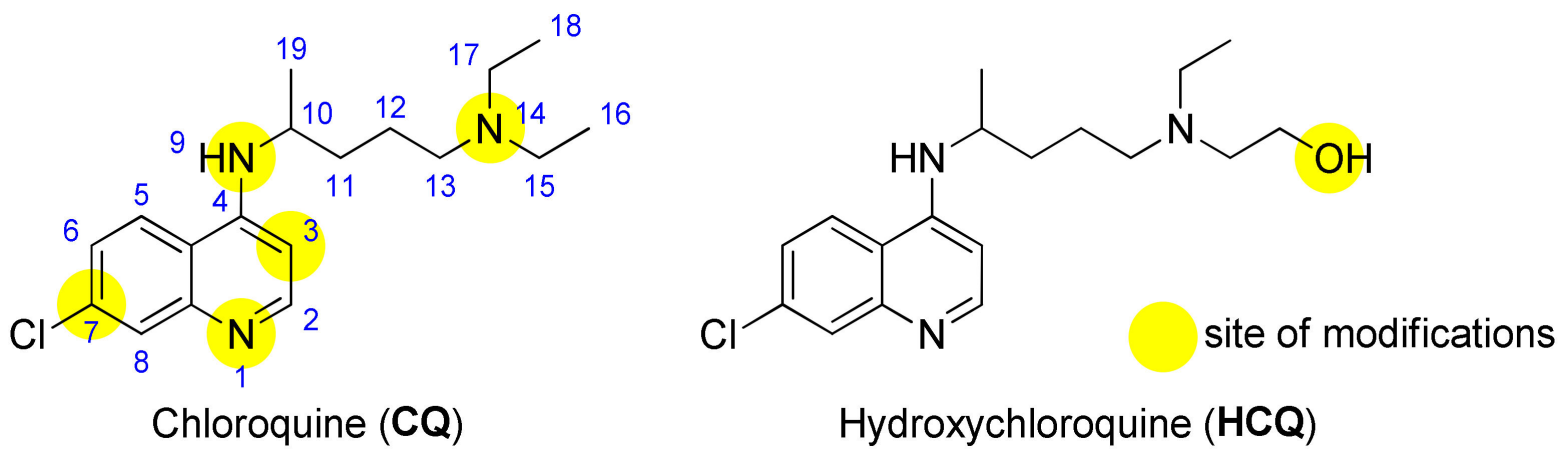
Structure, modification sites, and traditional atom numbering of chloroquine and hydroxychloroquine.

The reported modifications of the drug molecules include reactions at quinoline and diamine parts. The reactivity of the quinoline ring includes 7-chlorine nucleophilic substitution, electrophilic or radical substitution at position 3, and alkylation at the quinoline nitrogen atom. The side chain reactivity was exploited at both nitrogen atoms. Hydroxychloroquine displays additional reactivity of the primary alcohol, which seems to be an easy target for modification (Figure 5). Nevertheless, few such approaches can be found in the literature. Generally, due to the low complexity of the total synthesis of the drugs and rather limited reactivity of the remaining functional groups, a lot of analogs were de novo synthesized rather than adopting the top-down approach.

### 3.1. Quinoline

#### 3.1.1. N-1 Modifications

Alkylation at the quinoline nitrogen atom was performed in a reaction of chloroquine with a quaternary ammonium salt derived from ferrocene in the presence of K_2_CO_3_. Next, the reaction with l-(+)-tartaric acid caused the exchange of the anion. The product **CQ-1** exhibited lower antimalarial activity than the starting material in respect to every tested *P. falciparum* strain by up to an order of magnitude. It was implied that the compound lost the activity due to quaternization, preventing interactions with heme [88].


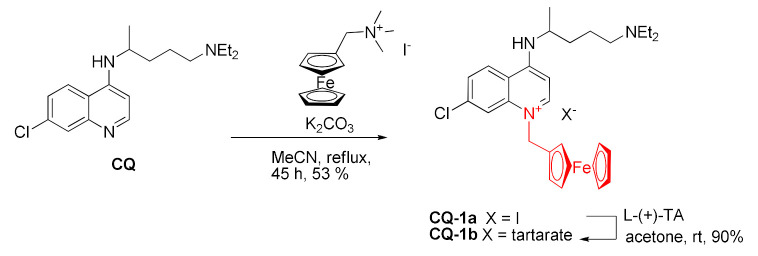


#### 3.1.2. C-3 Modifications

Chloroquine undergoes radical iodination at position 3 of the quinoline ring when treated with *N*-iodosuccinimide and trifluoroperoxyacetic acid. This modification decreased the antimalarial activity of **CQ-2** against *P. falciparum* (3D7, D10, Dd2, K1) by at least an order of magnitude compared to unmodified **CQ**. However, the synergistic effect of both compounds was observed [89].

Electrophilic substitution with tritium (^3^H) at position 3 was performed on chloroquine. For the reaction, fluorinated sulfonic acid catalyst supported on a polymer was effective, unlike some other Lewis (AlCl_3_) and weaker Bronsted acids (TFA). In the reaction, all chloroquine (**CQ** and **CQ-3**) was recovered [90]. Tritium labeling was conducted to help examine metabolic and excretory pathways.


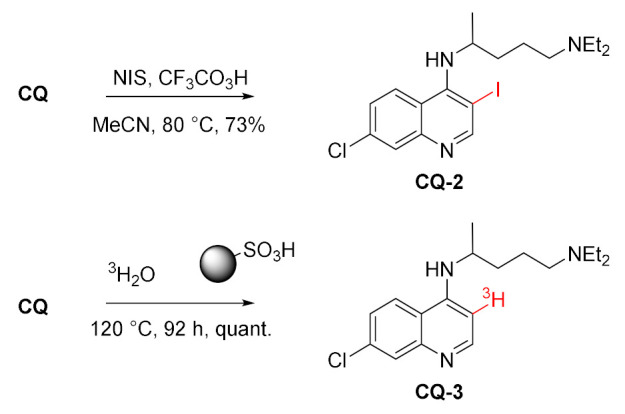


#### 3.1.3. C-7 Modifications

Susceptibility of chloroquinoline unit to the nucleophilic aromatic substitution at position 7 was exploited in the reactions with thioureas. This way, a series of 7-dechloro-7-thiourea derivatives was obtained. The thiourea **CQ-4a** with a free NH_2_ group was further reacted with a few isothiocyanates providing dithiobiurets **CQ-5a**–**e** in fair to good yields [91].


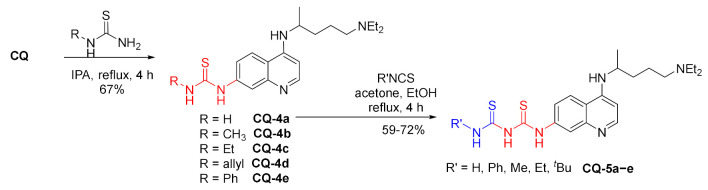


Lipshutz et al. developed a selective nickel-catalyzed method for the reduction of aryl halides. One of the substrates studied in this process was chloroquine, which was hydrodehalogenated at position 7 to give CQ-6 in a quantitative yield [92].


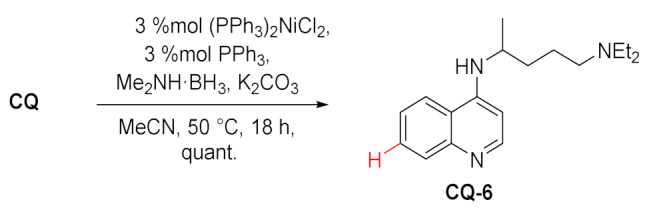


A similar reaction was performed with catalytic palladium *N*-heterocyclic carbene complex. With α-deuteriobenzhydrol as a stoichiometric reductant, the reaction proceeded in an excellent yield and resulted in 99% incorporation of deuterium [93].


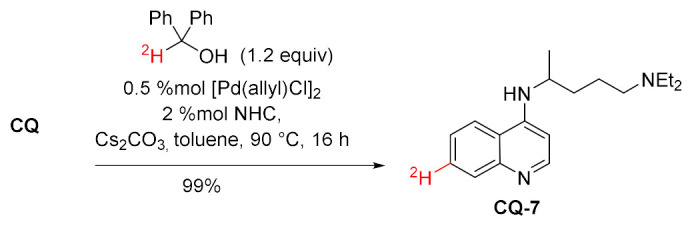


### 3.2. Aminoalkyl Part

#### 3.2.1. Secondary Amino Group

A set of carbamates decorated with bisphosphonate units was obtained from chloroquine. First, selected primary alcohols were treated with diphosgene to form reactive chloroformates. Then, reaction with either chloroquine or hydroxychloroquine gave the corresponding carbamates. In the last step, phosphonic esters were hydrolyzed to form bisphosphonic acid derivatives including **CQ-8** [94]. The compounds received in this way were assessed for the treatment of myeloma multiplex, a type of cancer in which delivery of the drug to bone is important. The bisphosphonate fragment helped to accumulate the drug in bones, where it could effectively prevent bone loss by killing myeloma cells. The compound **CQ-8** and the analogous **HCQ** derivative showed 70% killing of CD138^+^ myeloma cells, while a corresponding conjugate of bortezomib (a drug used to treat multiple myeloma) killed 65% of the cells. Additionally, the combined therapy with both conjugates was shown to kill 90% of the cells [95].


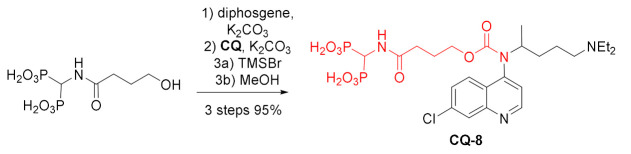


Amides were likely formed in the process of immobilizing chloroquine on gold nanoparticles. Carboxylic acid-coated nanoparticles were obtained by NaBH_4_ reduction of gold(III) chloride in the presence of 11-mercaptoundecanoic acid. Final coupling was performed using the carbodiimide method with EDC/*N*-hydroxysuccinimide resulting in an estimated 79% bonding. The conjugates were shown to interact with serum albumin [96], as well as presented some cytotoxicity against the MCF-7 tumor cell line by induction of autophagy. **CQ** was released from the nanoparticle at low pH [97].

#### 3.2.2. Tertiary Amino Group

Reactivity of the tertiary alkyl amine at position 14 was exploited in few reactions. Chloroquine was very efficiently and chemoselectively transformed into the corresponding hydrazinium salt **CQ-9**. The reaction relied on in situ generated iodonitrene intermediate from ammonium carbamate as a nitrogen source and iodosylbenzene as an oxidant [98].


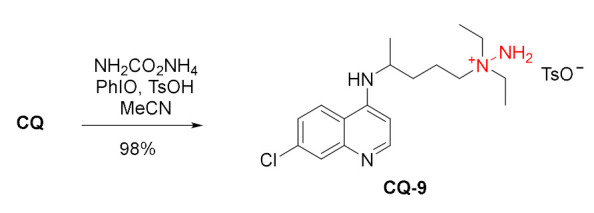


The application of 2,2,2-trichloroethyl chloroformate [99] to the two-step dealkylation of **CQ** resulted in rather efficient removal of one of the ethyl groups. Additional annulation product **CQ-10** was obtained from the reaction mixture [100]. Desethylchloroquine (**CQ-12**) was previously de novo synthesized in many steps [101].


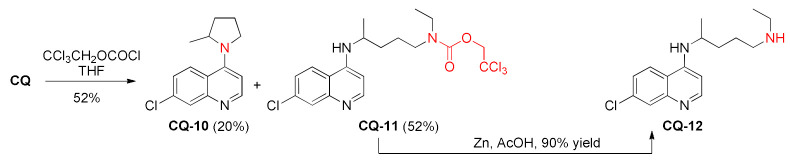


#### 3.2.3. Photochemical Degradation

A series of photochemical experiments was performed on **CQ**. Solutions of the drug were irradiated (240–600 nm), and seven products were identified. Two structures (**CQ-12** and **CQ-13**) are well-known metabolic products in vivo. In every product, the chlorine atom at C-7 was retained, even if the side chain was cleaved [102]. The same pattern was observed with hydroxychloroquine [46]. However, photodechlorination was possible under aerobic conditions [103]. Photochemical degradation of both chloroquine and hydroxychloroquine also produced the dimerization product **CQ-14** [46,104].


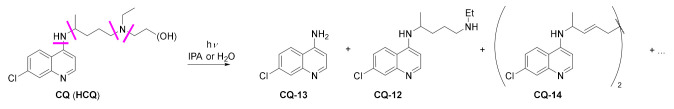


### 3.3. Reactions of Primary Alcohol Group of Hydroxychloroquine

#### 3.3.1. Etherification

A chemoselective oxa-Michael addition of **HCQ** hydroxy group to α,β-unsaturated sulfonyl and acryl ester was developed. The processes were catalyzed by silver diphosphine complex, a soft Lewis acid, and alkaline metals hexamethyldisilazanamides (HMDS) and Brønsted bases. In the reaction, the remaining amino groups did not react. The addition of **HCQ** to vinylsylfonamide gave sulfonamide ether **CQ-15** in an excellent yield [105], while the reaction with *tert*-butyl acrylate gave ester ether **CQ-16** in a rather poor yield [106].


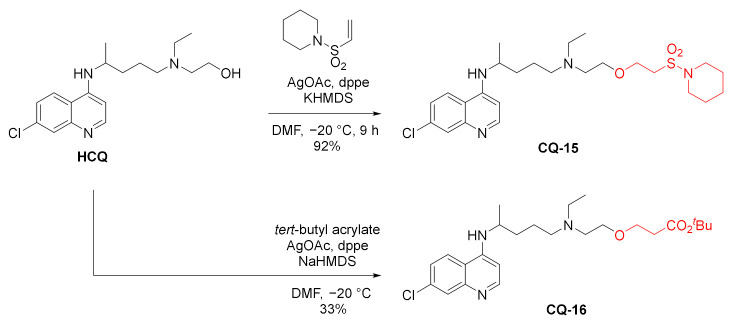


#### 3.3.2. Esterification

A few nonsteroidal anti-inflammatory drugs (NSAIDs) having carboxylic acid moiety were activated with carbonyldiimidazole and reacted with **HCQ** to yield corresponding esters **CQ-17a**–**f** in good yields. Both components are used separately in the treatment and management of rheumatoid arthritis and rely on different therapeutic mechanisms. The authors addressed problems with the unfavorable pharmacologic profile of **HCQ** and the side effects of NSAIDs in the gastric track by making a hydrolyzable link in the double prodrugs **CQ-17a**–**f**. The products did not undergo hydrolysis in the stomach (pH 1.2) but in the intestine (phosphate buffer pH 7.4). In terms of therapeutic activity, aceclofenac **CQ-17c** and licofelone **CQ-17e** prodrugs displayed the most promising anti-inflammatory response [107].


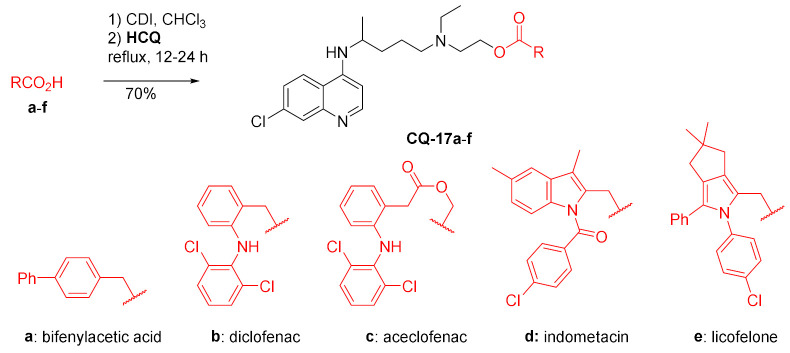


The carbodiimide method was used to produce the ester of hydroxychloroquine with hexanoic acid derivative of menadione in a fair yield. The acid is a potent glutathione reductase (GR) inhibitor (ED_50_ 3.5 µM). This enzyme is indirectly responsible for the resistance of *P. falciparum* to chloroquine. The enzyme increases the intracellular concentration of glutathione, which in turn protects malaria parasites from oxidative stress and stimulates heme catabolism. The conjugate drug **CQ-18** was nearly 2.5 times more active against *P. falciparum* FcB1R than unmodified **HCQ** with ED_50_ values of 107 and 259 nM, respectively [108].


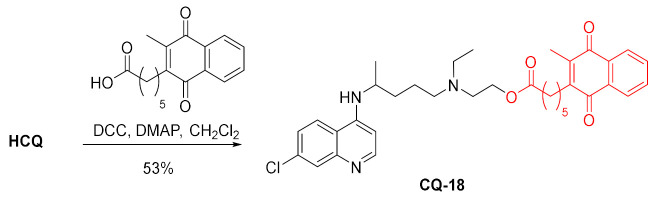


Oshima and coworkers developed a highly chemoselective transesterification method using µ-oxo-dinuclear iron(III) salen catalyst. One of the examples of alcohols was hydroxychloroquine, which gave the corresponding benzoate **CQ-19** in a very good yield [109].


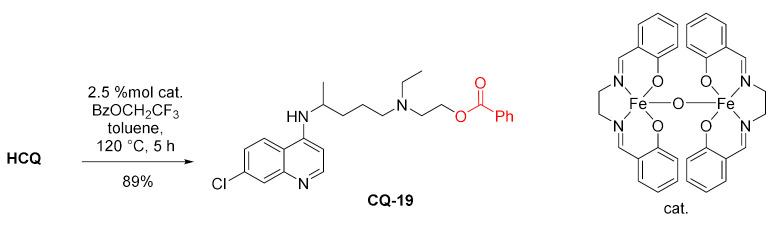


The hydroxy group of **HCQ** was also substituted for the amino group in a two-step process. First, hydroxychloroquine was converted to the chloroderivative **CQ-20** in the reaction with SOCl_2_. The chloroderivative **CQ-20** was treated either with ammonia in alcohol to form the primary amine **CQ-21** or with two small ring primary amines with another hidden amino group (3-amino-1-Boc-azetidine and Boc-bicyclo[1.1.1]pentane-1,3-diamine) to form the corresponding secondary amines **CQ-22** and **CQ-23**. Further acylation of the primary amine **CQ-21** was performed using uronium salt activation (*N*,*N*,*N*′,*N*′-tetramethyl-*O-*(benzotriazol-1-yl)uronium tetrafluoroborate, TBTU). Coupling partners acids with fluorinated and heterocyclic parts were used to give corresponding amides **CQ-24**–**25** in good yields [110].


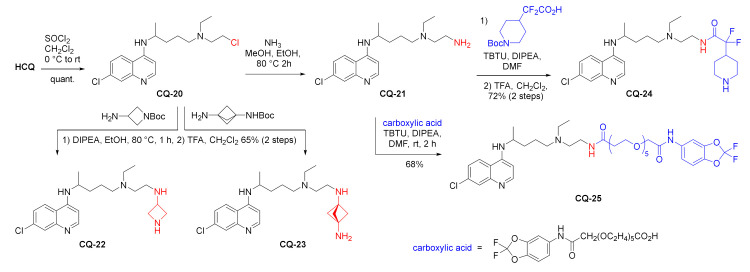


The introduced piperidine fragment in compound **CQ-24** was transformed in alkylation or acylation reactions into three further modified products **CQ-26**–**28** in 65–80% yields. The compounds were screened in the antiviral assay for COVID-19, showing at most marginal activity (5 µM for **CQ-27**). A more encouraging effect of the compounds **CQ-25**–**28** was found in their interaction with the immune system. All of them inhibited concanavalin A-induced T-cell proliferation and LPS-induced B-cell proliferation, and substantially inhibited cytokine production by these cells. The authors suggested potential for the compounds in the treatment of immune disorders and inflammatory diseases [110].


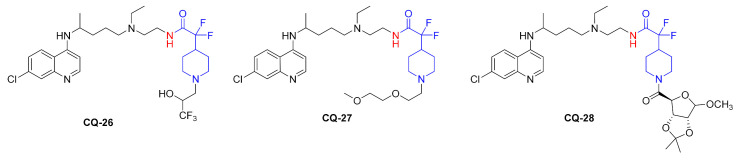


Modified polysaccharides such as hydroxyethyl starch (HES) are characterized by biocompatibility, biodegradability, and low immunogenicity. Therefore, they can be used as drug carriers. Hydroxychloroquine was conjugated to HES with the carbonate linker. In the first step, the active amide **CQ-29** was synthesized from **HCQ** and carbonyldiimidazole in a good yield. The subsequent reaction with HES gave polymeric materials **CQ-30** containing 2.4–32 w% of hydroxychloroquine. The yield was in the 61–82% range. The preserved secondary amino group was important for binding to the CXCR4 receptor in order to inhibit the spread of tumor cells. The polymer at 1 µM-**CQ** concentration inhibited pancreatic cancer cell invasion by approximately 80% (AsPC-1), 70% (MiaPaca-2), and 10% (MiaPaca-1). The unmodified drug remained inactive [111].


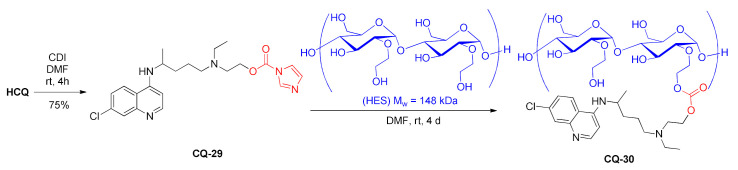


Hydroxychloroquine was used to prepare hydrolyzable and nondegradable methacrylamide copolymers. The reaction of methacryloyl chloride with **HCQ** gave the corresponding ester **CQ-31** in a moderate yield. Radical copolymerization of **CQ-31** with *N*-hydroxypropylmethacrylamide (HPMA) gave the polymeric material **CQ-32**. The polymer had *M*_W_ 34.5 kDa, a polydispersity index of 1.1, and 17 mol% chloroquine loading [112].


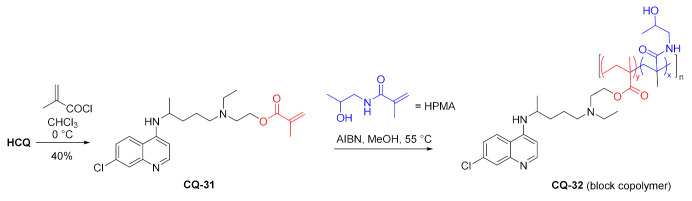


The hydroxy group of **HCQ** was substituted with azide in a Mitsunobu-type reaction with diphenylphosphorylazide and DBU (Merck method). The obtained azide **CQ-33** underwent copper(I)-catalyzed alkyne-azide 1,3-dipolar ‘click’ cycloaddition (CuAAC) with alkynes. The reaction with the *N*-propargyl-methacrylamide monomer gave the corresponding triazole **CQ-34**. Further RAFT copolymerization of **CQ-34** with HPMA produced the polymeric material **CQ-35A**. A similar product (**CQ-35B**) was produced in CuAAC of **CQ-33** with a polymethacrylamide polymer decorated with terminal alkyne groups. The polymers had comparable *M*_W_ 20.3 and 21.4 kDa, while the polydispersity index was 1.1 and 1.2, and chloroquine loading was 13.5 and 17.4 mol% for **CQ-35A** and **CQ-35B**, respectively. Both **CQ-32** and **CQ-35A/B** exhibited increased inhibition of 4T1 breast cancer cell migration in comparison with **HCQ**. The migration was decreased by 49% (**CQ-35A**), 51% (**CQ-35B**), and 56% (**CQ-32**), which proved that the effect can be obtained without chloroquine release and that the triazole ring did not interfere with the anticancer activity. The polymeric drugs also exhibited favorable cytotoxicities [112,113,114].


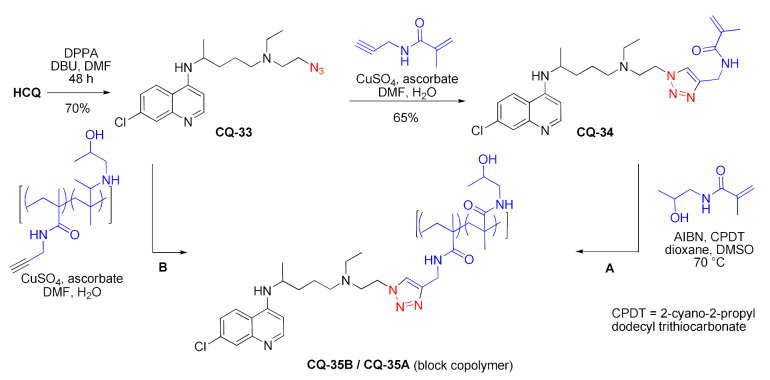


Multicomponent conjugates containing both gold nanoparticles, siRNA, PEG, and hydroxychloroquine were reported. The link between gold and **HCQ** was made by esterification of complexed citrate under carbodiimide activation. Hydroxychloroquine was found to improve transfection of cells by the RNA and accumulation of nanoparticles in endosomes [115].

#### 3.3.3. Miscellaneous

The electrochemical oxidative process, in which the hydroxy group of **HCQ** was probably oxidized to an aldehyde, was developed for analytical purposes. With multi-walled carbon nanotube-modified carbon paste electrode (MWCNTs/CPE) in the adsorptive stripping, differential pulse voltammetry nanomolar concentrations of pharmaceuticals in blood serum were determined [116].

### 3.4. Metal Complexes

There are a few known isolated coordination compounds of chloroquine with transition metal ions (Table 1).

**Figure 6:**
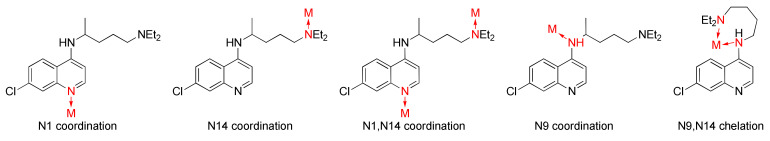
Coordination modes of chloroquine.

**Figure 7:**
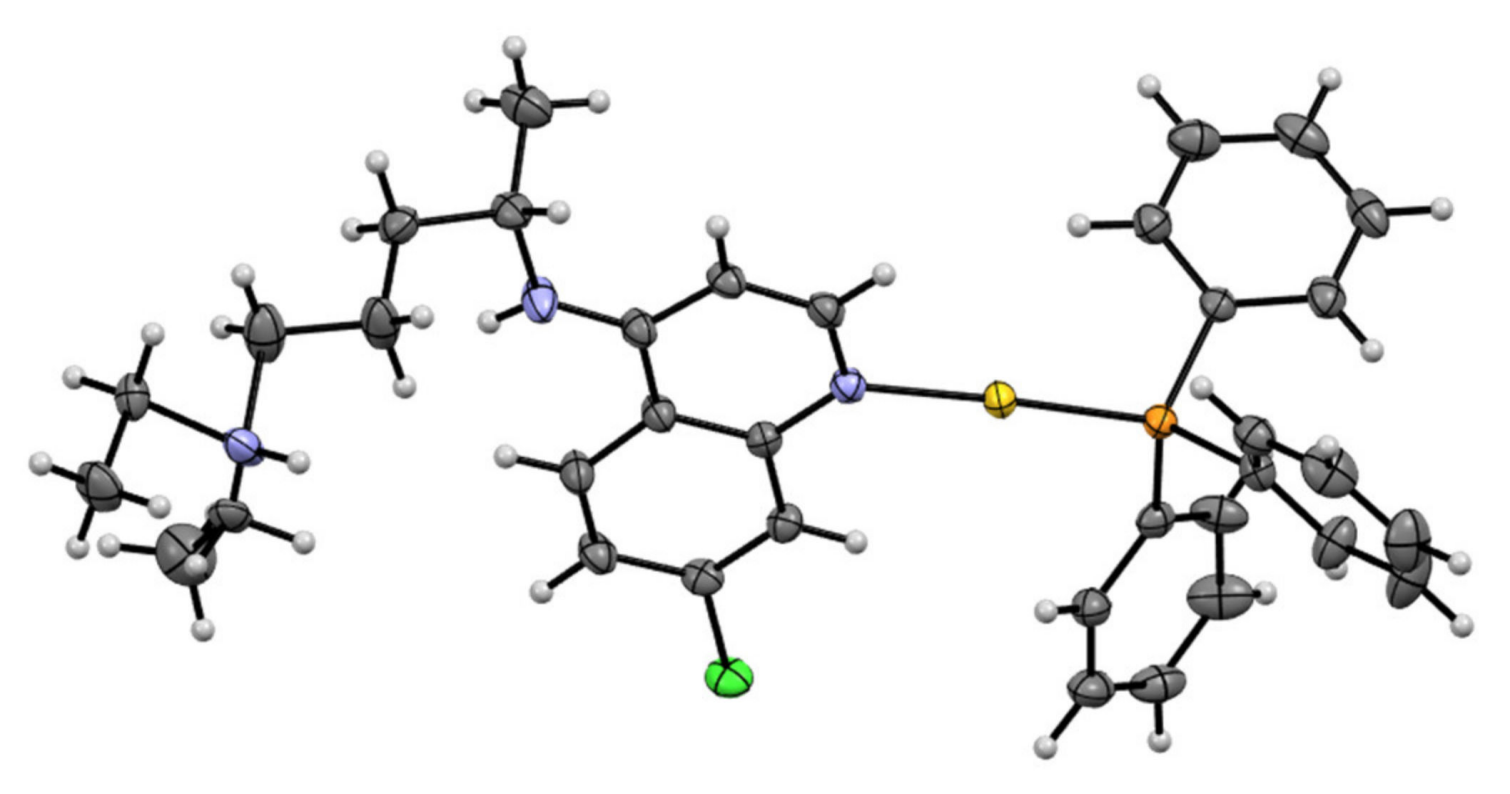
X-ray structure of [Au(PPh_3_)(chloroquine-H^+^)](NO_3_)_2_ [120].

Chloroquine possesses three nitrogen donor atoms capable of coordinating to a metallic center: the most common quinoline N-1 atom, tertiary alkyl amine N-14, and secondary arylamine N-9. The last one was observed in the complexes of multiply protonated chloroquine. Bidentate coordination has been reported either for two separate metallic centers or in a chelation mode (Figure 6). Moreover, η^6^ coordination to the benzene part of quinoline was postulated. Structure elucidation mostly relied on spectroscopic data (e.g., IR, NMR), only in one case X-ray structure provided unambiguous proof (Table 1, entry 1, Figure 7). Some discrepancies between reported coordination modes may exist (Table 1, entry 13). Most development has been made for complexes with gold, platinum, and ruthenium, which display improved antimalarial and antiproliferative properties.

A few gold complexes of chloroquine were prepared by Navarro and coworkers. Particularly, gold(I) complexes similar to [Au(PPh_3_)**CQ**]PF_6_ displayed activity against *P. falciparum* enhanced by an order of magnitude. For the FcB1 strain, IC_50_ was reported at 5 nM (47 nM for **CQ**) and for FcB2 strain 23 nM compared to 110 nM for **CQ**. In vivo study on the *P. berghei* model revealed lower parasitemia for the treatment with the complex (7%) compared to **CQ** (25%) [117]. Later tests revealed that both N-1 coordinated gold(I) and N-14 coordinated gold(III) complexes were active against multiple strains of *P. falciparum* (FcB1, W2, K1, F32) in vitro. The most active was [Au(PEt_3_)**CQ**]PF_6_ [118]. The X-ray structure of a related complex was published (Figure 7) [120]. Gold(I) was also used to tether two antimalarials, chloroquine and primaquine (PQ), into a single molecule. The resulting double drug exhibited higher activity against *P. falciparum* (3D7, W2) in vitro and *P. berghei* (liver-stage) in vivo than unlinked drugs [121].

Platinum(II) *cis-*complexes with phosphine ligands presented stronger interaction with DNA and serum albumin (BSA) than the unmodified drug. The bistriphenylphosphine-containing complex presented moderate cytotoxic activity against a few human tumor cell lines. The IC_50_ values were in the 5–10 µM range for breast (MDA-MB-231 and MCF-7), lung (A549), and prostate cancer cell lines (DU-145) [126]. Platinum **CQ** complexes were found to bind with DNA. The nature of interactions was dependent on the complex type, with N-14 coordinate *trans*-[Pt(**CQ**)_2_Cl_2_] displaying the most covalent character. This complex also displayed a cytostatic effect on multiple cancer cell lines. The growth inhibition, GI_50_ in the 6–9 µM range, and total growth inhibition at ≤19 µM were noted for human HT-29, LoVo, MCF-7, SKBR-3, PC-3, and murine B16/BL6 [127]. Liposome encapsulation of *trans*-[Pt(**CQ**·H_3_PO_4_)_2_Cl_2_] was performed [128].

Among complexes of chloroquine and ruthenium, [Ru(**CQ**)Cl_2_]_2_ displayed up to 4-fold increased effectiveness against chloroquine-resistant strains of *P. falciparum*, and *P. berghei* [125]. On the other hand, the arene diamine complex [Ru(η^6^-*p*-cymene)(4,7-diphenyl-1,10-phenanthroline)**CQ**](PF_6_)_2_ was found to interact with DNA and serum albumin, as well as noticeably inhibit tumor cell growth in vitro (A549, MDA-MB-231, MCF-7, L929) with IC_50_ in the 0.8–6 µM range [124].

Metal organic frameworks (MOF) with incorporated chloroquine were reported. Matrices based on zinc [137], zirconium [138], and titanium [139] were studied for the therapeutic release of **CQ** from the material.

### 3.5. Noncovalent Compounds

Supramolecular inclusion complexes of chloroquine with some common host molecules are known. For example, complexes with α- and β-cyclodextrins [140] and cucurbit [7] uril [141] all displayed 1:1 stoichiometry.

**Figure 8:**
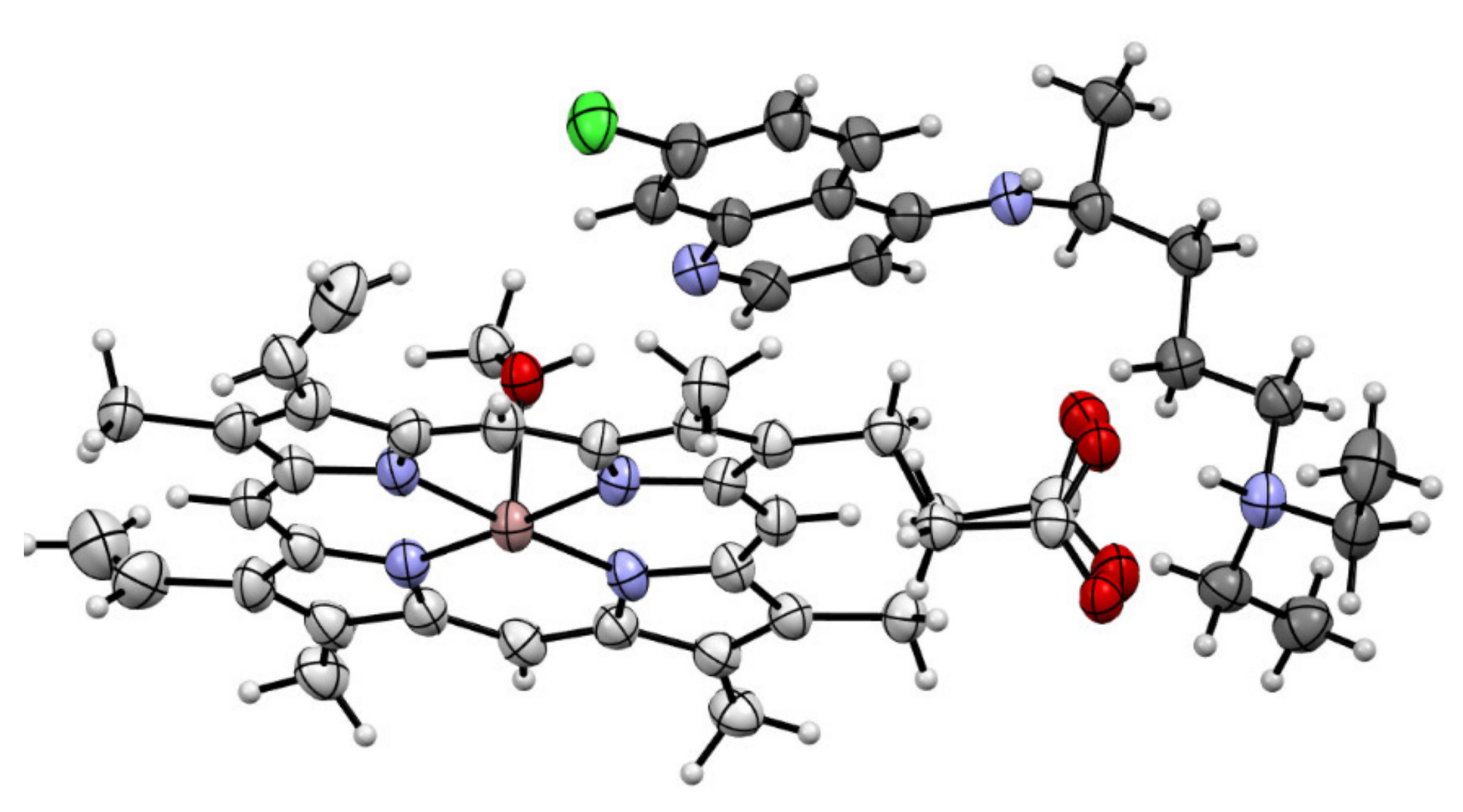
Part of X-ray structure of chloroquine: gallium(III) protoporphyrin IX compound [145].

A few attempts were made to identify compounds formed from chloroquine and various porphyrin/heme derivatives relevant to malaria. However, fully conclusive data was not obtained. A study on **CQ** interactions with ferriprotoporphyrin IX revealed stoichiometry of 1:2 CQ:Fe(III)PPIX, and dimerization via µ-oxo bond [142]. Ferrimesoporphyrin IX of mesohematin anhydride, a synthetic analog of hemozoin, was used to examine the mode of binding of chloroquine. EXAFS experiments indicated the proximity of the iron atom to quinoline N-1 and neighboring carbon atoms of chloroquine [143]. Non-covalent complexes of **CQ** with urohemin I (2:1 stoichiometry) and uroporphyrin were found in aqueous solutions [144]. Also in the X-ray crystal structure of gallium(III), protoporphyrin IX with **CQ** (2:2 stoichiometry) ionic and hydrogen-bonding interactions was found rather than covalent coordination links (Figure 8) [145].

Chloroquine was used to synthesize graphene oxide conjugates. Although the interactions are noncovalent (π-π interactions), there is some FT-IR evidence of binding between the amino group of chloroquine and carbonyl groups of graphene oxide. The nanoconjugates showed an antiproliferative effect on the A549 lung cancer cell line [146].

Chloroquine-containing chitosan-tripolyphosphate (CS-TPP) nanoparticles were prepared in order to investigate their protective effect on the liver in *P. berghei* NK65 infection. FT-IR spectroscopy indicated conjugation of chloroquine with CS-TPP and red blood cell surface. The chloroquine-loaded nanoparticles show a higher decrease of the apoptotic cells than chloroquine alone (61 vs. 25%) [147].

## 4. Closing Remarks

The viability of the late-stage modification to the structure of antimalarials seems to be directly related to the difficulty of the de novo synthesis. For example, virtually none of the thousands of derivatives of *Cinchona* alkaloids were obtained by the total synthesis. In contrast, out of thousands of analogs of chloroquine, just a few described above were prepared from commercially available chloroquine. The modification is more challenging than the synthesis due to the poor reactivity of the remaining groups. Mefloquine is an intermediate case since its synthesis is multistep and somewhat difficult. However, in subsequent modifications, neighboring group participation may be experienced and the stereochemistry requires consideration.

The obtained products were tested in a few different assays, which were outlined in this review, while a summary of available biological data for the covalent derivatives can be found in the Supplementary Table S1. Improved antimalarial potency was found for some gold complexes of **CQ** and conjugates with artemisinin. Few compounds were shown to act synergistically with parent drugs, possibly reversing resistance. Immunomodulatory effect of conjugates with anti-inflammatory drugs **CQ-17** showed promise in the experimental treatment of arthritis. On the other hand, incorporation of difluoromethylene and heterocyclic parts (**CQ-25**−**28**) gave rise to immunosuppressive activity. Conjugation to polymeric carriers (**CQ-30**–**35**) inhibited invasion of some cancer cells.

## Figures and Tables

**Table 1:** Known isolated coordination compounds of metal ions with chloroquine (**CQ**) and the established coordination site.

Entry	Metal	Complex	Site	Reference
1	Gold(I)	[Au(PR_3_)**CQ**]X	N1^1^	[117,118,119,120]
2		[Au**CQ**(C_5_F_5_)]	N1	[119]
3		[Au**CQ**(PQ*)]PF_6_	N1	[121]
4	Gold(III)	[Au(Et_2_O)**CQ**(SR*)Cl]Cl	N14	[118]
5		[Au(**CQ**)_2_(Cl)_2_]Cl	N14	[118]
6	Ruthenium(II)	[Ru(arene*)**CQ(**Cl)_2_]	N1	[122,123]
7		[Ru(cymene)(bipy*)**CQ**]	N1	[124]
8		[Ru(cymene)(**CQ**·2H_3_PO_4_)](BF_4_)_2_	π-benzene	[122]
9		[Ru(**CQ**)(Cl)_2_]_2_	N1,N14	[125]
10	Rhodium(I)	[Rh**CQ**(cod)Cl]	N1	[125]
11	Platinum(II)	*cis*-[Pt(diphosphine*)**CQ**Cl]	N1	[126]
12		*trans*-[Pt(**CQ**·2H_3_PO_4_)_2_(X)_2_]	N9	[127,128]
13		*trans*-[Pt(**CQ**)_2_(X)_2_]	N14 or N1	[127,129]
14	Palladium(II)	[Pd(**CQ**)_2_(X)_2_]	N1	[130]
15	Iridium(I)	[Ir(cod)**CQ**Cl]	N1	[131]
16		[Ir**CQ**(acetone)_2_]PF_6_	N9,N14	[131]
17	Iridium(III)	Ir_2_Cl_6_(**CQ**)	N9,N14	[131]
18	Cobalt(III)	[Co**CQ**(en)_2_Cl]Cl_2_	N1	[132]
19	Vanadium(IV)	[VO(acac)_2_(**CQ**·H_2_SO_4_)]	N1	[133]
20	Chromium(III)	Cr(NO)(CN)_2_(**CQ**·2H_3_PO_4_)(H_2_O)	N1,N9	[134]
21	Zinc(II)	[Zn**CQ**(H_2_O)(OAc)_2_]_2_	unspecified	[135]
22		[Zn**CQ**(H_2_O)_2_Cl_2_]	N1	[135]
23	Iron(II)	[Fe(**CQ**)_2_(H_2_O)]Cl_2_	N9,N14^2^	[136] ^2^
24	Copper(I)	[Cu(**CQ**)_2_]Cl	N1	[135]
25		[Cu(PPh_3_)**CQ**(NO_3_)]	N1	[135]
26	Copper(II)	[Cu**CQ**(OAc)_2_]_2_	unspecified	[135]
27		[Cu(**CQ**)_2_(H_2_O)]Cl_2_	N9,N14^2^	[136] ^2^
28	Nickel(II)	[Ni(**CQ**)_2_(H_2_O)]Cl_2_	N9,N14^2^	[136] ^2^

^1^ Based on X-ray structure; ^2^ limited supporting data; * SR = tetraacetyl-thioglucose; PQ = primaquine; arene = *p*-cymene or benzene; acac = acetylacetone; bipy = 2,2′-bipyridine, also: 1,10-phenanthroline; cod = 1,4-cyclooctadiene; diphosphine = dppp, dppf, also: 2×PPh_3_; en = ethylenediamine.

## Supplementary Materials

The following supporting information can be downloaded, Table S1. Summary of hydroxy(chloroquine) derivatives and available in vitro and in vivo experimental data.
