# Supplementary material for: A Review of Modifications of Quinoline Antimalarials: Mefloquine and (hydroxy)Chloroquine
Source: Molecules. 2022 Feb 2;27(3):1003. doi: 10.3390/molecules27031003 (PMC8838516; doi:10.3390/molecules27031003)
Supplement: Supplementary file 1 [file molecules-27-01003-s001.zip › molecules-1572683-supplementary.pdf]

Table S1. Summary of hydroxy(chloroquine) derivatives and available in vitro and in vivo experimental data

Compound	Activity type	Result	Ref.
**MQ**	Antimalarial: *P. berghei* in mice	active at 13 mg/kg	[66]
	Antimalarial in *vitro*: *P. falciparum*	W2, EC_{50} for MQ: 2.5 nM; F32-TEM, IC_{50}: 87 nM,	[38,49]
	Cytotoxicity on Vero Cells	CC_{50}: 21 µM	[49]
	Antituberculotic: *M. tuberculosis* H37Rv	MIC: 33 µM, hydrochloride: 25 µg/mL	[68,70,72]
	Antituberculotic: *M. tuberculosis* MDR	strain T113, MIC: 33 µM	[72]
**MQ-1**	Antimalarial: *P. berghei* in mice	Not active at 13 mg/kg	[66]
**MQ-2**	Antimalarial: *P. berghei* in mice	Not active at 13 mg/kg	[66]
**MQ-3**	n/d		
**MQ-4**	n/d		
**MQ-5**	n/d		
**MQ-6a-d**	n/d		
**MQ-7**	n/d		
**MQ-8**	Antimalarial: *P. berghei* in mice	As active as MQ	[19]
**MQ-9**	n/d		
**MQ-10**	n/d		
**MQ-11**	n/d		
**MQ-12**	n/d		
**MQ-13**	Antituberculotic *in vitro*: M. tuberculosis H37Rv	MABA, MIC: 31 µM, LORA, MIC: 61 µM, Vero cells, IC_{50}: 10 µM	[40]
**MQ-14a**	Antimalarial *in vitro*: *P. falciparum* W2	EC_{50}: 3.0 nM, IC_{50}: 17 nM	[38,39]
**MQ-14b**	Antimalarial *in vitro*: *P. falciparum* W2	IC_{50}: 74 nM	[39]

**MQ-14c**	Antimalarial *in vitro*: *P. falciparum* W2	IC_{50}: 24 nM	[39]
**MQ-14d**	Antimalarial *in vitro*: *P. falciparum* W2	IC_{50}: 24 nM	[39]
**MQ-15**	Antituberculotic *in vitro*: *M. tuberculosis* H37Rv	MABA, MIC: 29 μM, LORA, MIC: 21 μM, Vero cells, IC_{50}: 10 μM	[40]
**MQ-16**	n/d		
**MQ-17**	Antituberculotic *in vitro*: *M. tuberculosis* H37Rv	Inactive: MABA, LORA, cytotoxicity (Vero cells), IC_{50} > 128 μM	[40]
**MQ-18**	Antituberculotic *in vitro*: M. tuberculosis H37Rv	Inactive: MABA, LORA, cytotoxicity (Vero cells), IC_{50} > 128 μM	[40]
**MQ-19**	Antituberculotic *in vitro*: M. tuberculosis H37Rv	MABA, MIC: 30 μM, LORA, MIC: 25 μM, Vero cells, IC_{50} > 128 μM	[40]
**MQ-20**	Antituberculotic *in vitro*: M. tuberculosis H37Rv	MABA, MIC: 7 μM, LORA, MIC: 8 μM, Vero cells, IC_{50}: 50 μM	[40]
**MQ-21**	Antituberculotic *in vitro*: M. tuberculosis H37Rv	MABA, MIC: 63 μM, LORA, MIC: 94 μM, Vero cells, IC_{50}: 98 μM	[40]
**MQ-22**	Antituberculotic *in vitro*: M. tuberculosis H37Rv	MABA, MIC: 22 μM, LORA, MIC: 31 μM, Vero cells, IC_{50}: 35 μM	[40]
**MQ-23**	Antituberculotic *in vitro*: M. tuberculosis H37Rv	MABA, MIC: 15 μM, LORA, MIC: 22 μM, Vero cells, IC_{50}: 38 μM	[40]
**MQ-24**	Antimalarial *in vitro*: *P. falciparum*	IC_{50}: 15.7 (F32), 12.7 (Thai), 17.2(FcB1), 10.6 (K1) nM	[34]
	Antimalarial *in vivo*: *P. berghei* NK173 in mice	more efficient in controlling the parasitemia than artemether	[34]
**MQ-25**	Antimalarial *in vivo*: *P. berghei* in mice	As active as MQ, no contractual effect on the isolated mouse diaphragm and no inhibition of directly simulated twitch response	[66,42]
**MQ-26**		no contractual effect on the isolated mouse diaphragm and no inhibition of directly simulated twitch response	[42]
**MQ-27**	n/d		
**MQ-28**	n/d		
**MQ-29**	n/d		
**MQ-30**	n/d		
**MQ-31**	n/d		
**MQ-32**	n/d		

**MQ-33**	Antimalarial *in vivo*: *P. berghei* in mice	Inactive	[66]
**MQ-34a**	n/d		
**MQ-34b**	Data available elsewhere	Jauch, R.; Griesser, E.; Oesterhelt, G. Metabolismus von Ro 21-5998 (Mefloquin) bei der Ratte, *Arzneimittelforschung* **1980**, *30*, 60-67.	
**MQ-35**	n/d		
**MQ-36**	n/d		
**MQ-37**	n/d		
**MQ-38**	Antimalarial *in vitro*: *P. falciparum*	F32-TEM, IC_{50}: 0.6 nM	[49]
	Cytotoxicity	Vero Cells, CC_{50}: 4 µM	
**MQ-39**	Antimalarial *in vitro*: *P. falciparum*	F32-TEM, IC_{50}: 1.1 nM	
	Cytotoxicity	Vero Cells, CC_{50}: 5 µM	
**MQ-40**	n/d		
**MQ-41**	Antimalarial *in vitro*: *P. falciparum*	IC_{50}: 6.6 (F32), 4.5 (Thai), 5.4 (FcB1), 2.4 (K1) nM	[34]
	Antimalarial *in vivo*: *P. berghei* NK173 in mice	highly effective in inhibiting the parasite growth (much more efficient than its CF_3-artemisinin derivative precursor); more efficient in controlling the parasitemia than artemether	
**MQ-42**	n/d		
**MQ-43**	n/d		
**MQ-44**	n/d		
**MQ-45**	n/d		
**MQ-46**	n/d		
**MQ-47**	Human adenosine A_{2A} receptor antagonist		[65]
**MQ-48**	Antimalarial *in vivo*: *P. vinckei petteri* in mice	inactive	[67]

**MQ-49**	Antimalarial *in vivo*: *P. vinckei petteri* in mice	inactive	[67]
**MQ-50**	Antimalarial *in vivo*: *P. berghei* in mice	Not active at 13 mg/kg	[66]
**MQ-51**	n/d		
**MQ-52**	n/d		
**MQ-53**	n/d		
**MQ-54**	n/d		
**MQ-55**	n/d		
**MQ-56**	n/d		
**MQ-57**	n/d		
**MQ-58**	n/d		
**MQ-59**	n/d		
**MQ-60**	n/d		
**MQ-61**	n/d		
**MQ-62**	n/d		
**MQ-63**	n/d		
**MQ-64a-q**	Antituberculotic: *M. tuberculosis* H37Rv	MIC: 11.9 μM (**MQ-64h**) to > 250 μM	[68,70]
	Antituberculotic: *M. tuberculosis* MDR	strain T113, MIC: 11.9 μM to 99.8 μM	[70]
**MQ-65**	n/d		
**MQ-66a-b**	Human adenosine A_{2A} receptor antagonist		[65]
**MQ-67a-b**	Human adenosine A_{2A} receptor antagonist		[65]
**MQ-68**	Antituberculotic: *M. tuberculosis* H37Rv	MABA, MIC > 128 μM, LORA, MIC > 128 μM	[40]
**MQ*HBPh$_4$**	Antituberculotic: *M. tuberculosis* H37Rv	MIC: 12 μg/mL	[72]
**MQ-69**	Antituberculotic: *M. tuberculosis* H37Rv	MIC: 50 μg/mL	[72]

**MQ-70**	Human adenosine A_{2A} receptor antagonist		[65]
**MQ-71**	Human adenosine A_{2A} receptor antagonist		[65]
**HCQ**	Antimalarial: *P. falciparum*	ED_{50} 107 nM (FcB1R)	[108]
**CQ-1**	Antimalarial: *P. falciparum*	IC_{50} 143-789 nM	[88]
**CQ-2**	Antimalarial: *P. falciparum*	IC_{50} 300-700 nM, synergistic effect with CQ	[89]
**CQ-3**	n/d		
**CQ-4a-e**	n/d		
**CQ5a-e**	n/d		
**CQ-6**	n/d		
**CQ-7**	n/d		
**(H)CQ-8**	Anticancer: drug-resistant multiple myeloma	Combined bortezomib therapy effective at 20 μM	[95]
**CQ-9**	n/d		
**CQ-10**	n/d		
**CQ-11**	n/d		
**CQ-12**	Data available elsewhere	Fu, S.; Björkman, A.; Wåhlin, B.; Ofori-Adjei, D.; Ericsson, O.; Sjöqvist, F. In Vitro Activity of Chloroquine, the Two Enantiomers of Chloroquine, Desethylchloroquine and Pyronaridine against Plasmodium Falciparum. *Br. J. Clin Pharmacol* **1986**, *22*, 93–96.	
**CQ-13**	Data available elsewhere		
**CQ-14**	n/d		
**CQ-15**	n/d		
**CQ-16**	n/d		
**CQ17a-f**	Antiinflammatory: rat	Carrageenan-induced paw edema: reduction 21-64% (1h), 55-84% (3h)	[107]
	Analgesic activity	Acetic acid -induced writhing method: 4-39% (1h), 73-83% (3h)	

	Anti-arthritic	CFA-induced arthritis 64-76% joint diameter reduction on 28th day	
**CQ-18**	Susceptibility to hydrolysis	$t_{1/2}$ > 80 h (pH 5, pH7.4, RPMI-10% human serum)	[108]
	Antimalarial: *P. falciparum*	ED_{50} 107 nM (FcB1R)	
**CQ-19**	n/d		
**CQ-20**	n/d		
**CQ-21-28**	SARS-Cov2 spike inhibition	CC_{50} and EC_{50} 5 μM (**CQ-27**) - 40 μM	[110]
	Cytotoxicity: mouse splenocytes	CC_{50} 6-70 μM	
	Immunosuppression	Con A-induced T-cell proliferation IC_{50} 1.3 μM (**CQ-25**) – 45 μM<br>LPS-induced B-cell proliferation IC_{50} 1.1μM (**CQ-25**) - 28 μM<br>Inhibition of INF-gamma secretion IC_{50} 0.35 μM (**CQ-28**) – 1.2 μM, IL-17 IC_{50} 0.17 μM (**CQ-28**) – 2 μM,<br>IL-6(Con-A stimulated) IC_{50} 0.16 μM (**CQ-26**) – 3 μM,<br>IL-6(LPS stimulated) IC_{50} 0.01 μM (**CQ-26**) – 1.5 μM,	
**CQ-29**	n/d		
**CQ-30**	cytotoxicity	>300 μM(CQ)	[111]
	Study of action	Autophagy inhibiyion, via CXCR4 pathway	
	Cancer cell migration:	70% inhibition at 1 μM(CQ) for Mia Paca-2, 10 μM(CQ) for Mia Paca-1, <1 μM(CQ) for AsPC-1	
**CQ-31**	n/d		
**CQ-32**	Cancer cell migration:	4T1, 50% inhibition at 30 μM(CQ)	[114]
		4T1, Antimetastatic effect in vivo	[113]
	Study of action	Inhibition of CXCR4, CXCL12, and SDF-1 chemokine axis, translocation to cytoplasm, antimetastatic effect	[113]<br>[112]

	Cytotoxicity	Jurkat, Taji >100 μM(CQ)	[112]
**CQ-33-34**	n/d		
**CQ-35**	Cancer cell migration:	4T1, 50% inhibition at 30 μM(CQ)	[114]

n/d - the compound has not been evaluated in biological assay or such data has not been reported
